# Microbiome differential abundance methodologies to detect relevant taxa associated with chemotherapy toxicity rate in colorectal cancer

**DOI:** 10.1093/bioadv/vbag148

**Published:** 2026-06-24

**Authors:** Elsa Martín-De Arribas, Kelly Conde-Pérez, Pablo Aja-Macaya, Juan A Vallejo, Germán Bou, Ana López-Cheda, María Amalia Jácome-Pumar, Susana Ladra, Margarita Poza

**Affiliations:** Universidade da Coruña, CITIC, Database Laboratory, A Coruña, 15071, Spain; Microbiology Research Group, Institute of Biomedical Research (INIBIC), Interdisciplinary Center for Chemistry and Biology (CICA)—University of A Coruña (UDC)—CIBER de Enfermedades Infecciosas (CIBERINFEC-ISCIII), A Coruña, 15006, Spain; Microbiology Research Group, Institute of Biomedical Research (INIBIC), Interdisciplinary Center for Chemistry and Biology (CICA)—University of A Coruña (UDC)—CIBER de Enfermedades Infecciosas (CIBERINFEC-ISCIII), A Coruña, 15006, Spain; Microbiology Research Group, Institute of Biomedical Research (INIBIC), Interdisciplinary Center for Chemistry and Biology (CICA)—University of A Coruña (UDC)—CIBER de Enfermedades Infecciosas (CIBERINFEC-ISCIII), A Coruña, 15006, Spain; Microbiology Research Group, Institute of Biomedical Research (INIBIC), Interdisciplinary Center for Chemistry and Biology (CICA)—University of A Coruña (UDC)—CIBER de Enfermedades Infecciosas (CIBERINFEC-ISCIII), A Coruña, 15006, Spain; Universidade da Coruña, CITIC, Grupo de Modelización, Optimización e Inferencia Estatística (MODES), Departamento de Matemáticas, Facultade de Informática, A Coruña, 15071, Spain; Universidade da Coruña, CITIC, Grupo de Modelización, Optimización e Inferencia Estatística (MODES), Departamento de Matemáticas, Facultade de Informática, A Coruña, 15071, Spain; Universidade da Coruña, CITIC, Database Laboratory, A Coruña, 15071, Spain; Microbiology Research Group, Institute of Biomedical Research (INIBIC), Interdisciplinary Center for Chemistry and Biology (CICA)—University of A Coruña (UDC)—CIBER de Enfermedades Infecciosas (CIBERINFEC-ISCIII), A Coruña, 15006, Spain; Universidade da Coruña, Microbiome and Health Research Group, Departamento de Biología, Facultade de Ciencias, A Coruña, 15071, Spain

## Abstract

**Motivation:**

The interplay between microbial communities and treatment outcomes represents a promising area in pharmacomicrobiomics. Identifying microbial biomarkers that differentiate toxicity levels could inform personalized cancer strategies. However, biomarker identification is strongly influenced by methodological choices in differential abundance analysis (DAA), and most studies focus on individual outcomes despite toxicity being inherently multifactorial. In this study, we defined a multi-dimensional toxicity variable integrating clinical symptoms and treatment modifications to stratify colorectal cancer patients. We then evaluated six widely used DAA methods (ALDEx2, ANCOM-BC, DESeq2, LEfSe, LinDA, and ZicoSeq) to assess how analytical variability affects the detection of microbiome signatures associated with chemotherapy-related toxicity. Analyses were performed under different preprocessing and multiple-testing correction strategies, and consistency was further examined using an independent validation dataset.

**Results:**

Substantial variability was observed across methods, with limited overlap in detected taxa but moderate concordance in effect-size rankings. ANCOM-BC showed the most consistent overall performance across analytical scenarios, although trade-offs remained between taxa detection, ranking, and direction of association. Despite this variability, a subset of taxa was consistently identified across methods, including *Parvimonas*, *Eubacterium ventriosum group*, and *Ruminococcus* in the low-toxicity group, and members of the *Lachnospiraceae* family, such as *Fusicatenibacter*, *Lachnospira*, and the *Lachnospiraceae NK4A136 group*, in the severe-toxicity group. Analyses in the external validation dataset supported the reproducibility of methodological patterns, despite differences in cohort composition and sequencing strategy. These findings highlight the methodological dependence of microbiome biomarker discovery and the potential of pre-treatment microbial signatures to stratify toxicity risk. View collectively, our results support a context-dependent approach to DAA method selection in clinical microbiome studies.

**Availability and implementation:**

The data supporting this study are available at NCBI SRA database (PRJNA911189) and NCBI SRA database (PRJNA893853).

## 1 Introduction

The human microbiome is a diverse community of microorganisms that live in our bodies and has recently garnered significant attention due to its impact on health and disease (Berg *et al.* 2020, Hou *et al.* 2022). Microbial imbalance, also known as dysbiosis, is associated with diseases such as colorectal cancer (CRC), underscoring the importance of understanding the microbiome’s composition and its impact on disease progression. Recently, growing concern has focused on the bidirectional interactions between the microbiome and anticancer drugs (Lee *et al.* 2021, Dougherty and Jobin 2023, Zhao *et al.* 2023). Consequently, rigorous statistical frameworks are required to unravel these medication-induced shifts.

Analysing microbiome data requires novel approaches due to its unique complexities. Differential abundance analysis (DAA) methods are essential for identifying taxa that differ significantly across experimental groups or conditions, thereby enhancing our understanding of the microbiome’s role in health (Deng *et al.* 2018, Li *et al.* 2020, Wallen 2021, Nearing *et al.* 2022, Yang and Chen 2022, Calgaro *et al.* 2023, Wirbel *et al.* 2024, Pelto *et al.* 2025). However, these methods face specific challenges, including zero inflation (resulting in highly sparse matrices), variability, and compositionality (since only relative abundances are observed and constrained to a constant sum).

To mitigate these challenges, recent DAA pipelines incorporate tailored normalization and preprocessing steps. A key question in microbiome data analysis is whether to rarefy (subsample) the data to standardize read depths across samples (Weiss *et al.* 2017). This practice has faced criticism for potentially discarding valuable samples and increasing false positives (McMurdie and Holmes 2014). Consequently, DAA methodologies have adopted various normalization and preprocessing steps to address these issues (Segata *et al.* 2011, Fernandes *et al.* 2014, Lin and Peddada 2020a,b, Cappellato *et al.* 2022, Nearing *et al.* 2022, Yang and Chen 2022, 2023). For instance, some studies rarefied the input for LEfSe before transforming it into relative abundances (Nearing *et al.* 2022), while others did not (Wallen 2021, Swift *et al.* 2023).

This research aims to provide a comprehensive review of commonly used DAA methodologies, tested on a real-world dataset comprised exclusively of a North-Western Spanish cohort of CRC patients, rather than comparing controls to patients. By focusing on a geographically homogeneous cohort, we minimize potential confounding arising from diet and environmental exposures. These patients were classified into two toxicity-ranked groups based on the secondary effects of chemotherapy treatment and treatment dose reductions. Against this backdrop, we benchmarked six representative DAA methodologies spanning count-based, compositional, linear-model, and zero-inflated paradigms. The selected DAA methods include ALDEx2, ANCOM-BC, DESeq2, LEfSe, LinDA, and ZicoSeq. Two approaches were evaluated: the first involving the use of the full dataset (unfiltered), while the second filtering taxa by prevalence. For each strategy, feature selection was performed with and without Benjamini–Hochberg (BH) adjustment to evaluate the control of the false discovery rate (FDR).

Through this study framework, the following research questions (RQs) will be addressed:

- RQ1: Would there be similarities among the DAA methodologies’ outputs applying different microbiome data transformations?
- RQ2: Would there be similarities among the DAA methodologies’ outputs with and without previous prevalence filtering criteria?
- RQ3: Could it be possible to identify differential features in abundance between the two toxicity groups?

The benchmarked methods were selected to represent both commonly used and recently proposed DAA methods in microbiome research, representing distinct methodological paradigms, from early marker-based techniques to compositional and regression-based models, thereby enabling a comprehensive evaluation in toxicity prediction and microbiome classification tasks.

## 2 Methods

### 2.1 Research design

To accomplish the aims of this investigation, six DAA methods were selected to identify taxa associated with chemotherapy toxicity in CRC patients. These methods were chosen to represent different statistical frameworks and abundance transformations commonly used in microbiome studies. The selected methods were applied to a homogeneous cohort of CRC patients receiving oxaliplatin (OX) and 5-fluorouracil (5-FU) chemotherapy (FOLFOX-like regimen). Microbiome profiles derived from 16S rRNA sequencing were integrated with clinical metadata to evaluate associations between microbial taxa and treatment-related toxicity.

We introduce a novel chemotherapy toxicity variable derived from an integrated assessment of multiple clinical symptoms typically associated with chemotherapy-related adverse effects, in conjunction with data on chemotherapy dose reductions and treatment interruptions. This primary outcome was operationalized as a dichotomous binary variable (low versus severe toxicity), hereafter referred to as toxicity. The analytical workflow involved applying each DAA method to an identical microbiome dataset and subsequently comparing their performance in identifying bacterial taxa significantly associated with toxicity. Comprehensive information on the study cohort, sequencing procedures, and accompanying metadata is provided in Subsection Dataset Description and in Table 1, available as supplementary data at *Bioinformatics Advances* online.

#### 2.1.1 Dataset description

This study analysed a publicly available dataset previously described by Conde-Pérez *et al.* (2024a,b). The original cohort consisted of 95 adults with histologically confirmed CRC, recruited between October 2017 and April 2021 at the University Hospital Complex of A Coruña (Spain). A single faecal sample was obtained from each participant before initiation of the first cycle of adjuvant chemotherapy and prior to any antibiotic exposure. The administration of antibiotics and probiotics was not permitted during the 4 weeks preceding sample collection.

For the present analysis, only patients treated with a FOLFOX-like chemotherapy regimen comprising OX and 5–FU, with or without concomitant radiotherapy, were included. Clinical metadata were extracted from the electronic medical records and integrated with the corresponding microbiome profiles. The primary endpoint was chemotherapy-related toxicity, operationalized as a binary variable: low toxicity (n=11) versus severe toxicity (n=25). Severe toxicity was defined according to the Common Terminology Criteria for Adverse Events v5.0 and required the occurrence of adverse events leading to a ≥20% dose reduction or discontinuation of 5-FU and/or OX.

The microbiome dataset, therefore, comprises 36 paired-end faecal samples sequenced on the Illumina MiSeq platform (2 × 300 bp), targeting the V3-V4 hypervariable region of the 16S rRNA gene. The sequencing data are publicly accessible in the NCBI Sequence Read Archive (SRA) under accession numbers PRJNA911189 and PRJNA893853. The study protocol was approved by the Galician Research Ethics Committee (CEIm-G; references 2018/609 and 2024/077, Spain) and by the Spanish Agency for Medicines and Healthcare Products (AEMPS) for the use of CRC patient samples from the University Hospital of A Coruña (CHUAC, A Coruña, Galicia, Spain). The study was conducted in accordance with the principles of the Declaration of Helsinki. Written informed consent for sample collection and storage in the CHUAC Biobank (A Coruña, UNE-EN ISO 9001:2015 certified) was obtained from all participants through the Servizo Galego de Saúde (SERGAS). Additional information on baseline characteristics and variable coding rules is provided in Table 1, available as supplementary data at *Bioinformatics Advances* online.

#### 2.1.2 Limitations

This study relies on a single real-world dataset comprising 36 CRC patients. The cohort is relatively small and unbalanced with respect to the toxicity outcome, with 11 patients in the low-toxicity group and 25 in the severe-toxicity group. An additional imbalance is observed in the sex distribution: the low-toxicity group comprises 9 males and 2 females, whereas the severe-toxicity group comprises 14 males and 11 females. Moreover, historical clinical records do not consistently specify the use of leucovorin or the exact oxaliplatin dose administered, which limits the level of treatment detail available for downstream analyses.

Finally, the dataset lacks a ground truth for toxicity-associated microbial biomarkers, which constrains the ability to objectively assess the performance of differential abundance methods. In addition, the identified associations have not been experimentally validated. While our results are statistically robust and supported by existing literature, the absence of functional validation limits the ability to establish causal relationships.

#### 2.1.3 External validation

For external validation, we considered the dataset described in Hillege *et al.* (2025), as, to the best of our knowledge, no other publicly available datasets meet the inclusion criteria required to reproduce this study’s analytical design. That dataset reports detailed symptomatology in CRC patients treated with capecitabine (CAP), an oral prodrug of 5-FU, together with prospectively collected information on chemotherapy dose modifications at multiple time points (baseline, during treatment, and post-treatment). Among the 56 CRC patients originally enrolled, symptom records were incomplete for a subset of individuals; consequently, only three symptoms [Fatigue, Hand–Foot Syndrome (HFS), and Peripheral Sensory Neuropathy (PSN)], together with dose delay or reduction events, were retained for analysis based on their clinical relevance and potential association with microbiome composition. To ensure comparability with our primary dataset, only baseline (pretreatment) microbiome profiles were evaluated against post-treatment symptom and dose delay information, and missing entries for the selected symptoms were imputed as 0.

Following the toxicity definition used in our study, a patient was classified as experiencing severe toxicity if any dose delay or reduction occurred or if at least one of the three selected symptoms reached grade ≥2; otherwise, the patient was categorized as having low toxicity. Applying these criteria resulted in a validation cohort of 48 patients (14 severe toxicity and 34 low toxicity), and, accordingly, the external validation analysis was restricted to the toxicity outcome. In addition, an important methodological difference between datasets is that Hillege *et al.* (2025) employed whole-metagenome shotgun sequencing with taxonomic profiling based on MetaPhlAn4, whereas our primary dataset relies on 16S rRNA sequencing; nevertheless, in both studies, only baseline microbiome profiles were used to assess associations with post-treatment toxicity, thereby ensuring consistency in the analytical framework.

#### 2.1.4 Sample-size justification

A power analysis was conducted using G*Power (v. 3.1.9.7) to determine the sample size required to detect a statistically significant difference in abundance between two independent groups (Faul *et al.* 2009, Kang 2021). Assuming a significance level α=0.05 and a desired statistical power 1−β=0.80, a total sample size of 11 (low-toxicity) and 25 (severe toxicity) was deemed sufficient to detect an effect size d=1.04. While this does not ensure high power for every feature tested, it supports the feasibility of identifying strong signals within our dataset.

#### 2.1.5 Dataset preprocessing

The analysis was initiated from the phyloseq object generated in our previous work (Conde-Pérez *et al.* 2024a,b). The initial cohort was restricted to participants who met the predefined eligibility criteria. Subsequently, we implemented pre-filtering procedures, including contaminant removal, following the recommendations of Salter *et al*. (2014). This denoising and contaminant-removal workflow yielded 6082 amplicon sequence variants (ASVs) prior to prevalence filtering, which were imported into R (v.4.4.0) via the RStudio integrated development environment (v.2024.4.1.748) (RStudio Team 2020).

To assess the impact of prevalence filtering, we analysed both the full ASV table and a prevalence-filtered variant that retained only ASVs detected in at least 5% of samples, resulting in 1045 ASVs. Each differential abundance (DAA) method was, therefore, executed under four input conditions [unfiltered versus prevalence-filtered and unadjusted versus Benjamini–Hochberg (BH)-adjusted], with microbiome data transformation selected in accordance with each method’s methodological recommendations. We adhered to the specific input requirements of each DAA approach; comprehensive implementation details are available in the accompanying GitHub repository. For each DAA tool, we report both raw and BH-adjusted *P-*value (Benjamini and Hochberg 1995). Because LEfSe typically outputs only a linear discriminant analysis (LDA) score, we applied BH correction to its associated *P-*value using the same adjustment function, thereby ensuring consistent control of the FDR across all methods.

We then performed two complementary analyses under multiple preprocessing and statistical configurations (Table 1). First, phyloseq feature tables were aggregated at the genus level using each tool’s native aggregation functionality (with the exception of ALDEx2 and DESeq2, which were provided with a pre-aggregated genus-level phyloseq object). Second, we conducted ASV-level analyses without taxonomic aggregation. The ASV-level analysis preserved maximal sequence resolution and maintained taxonomic annotations from phylum to species, where available. Species-level assignments are frequently unobtainable from 16S rRNA gene data and were, therefore, used exclusively for annotation and *post hoc* summarization (Johnson *et al.* 2019). Analysing data at the native ASV level can reduce signal conflation that may occur when heterogeneous features are merged into higher taxonomic ranks, whereas genus-level aggregation can partially alleviate sparsity. Given that the chosen taxonomic resolution can substantially influence the set of detected features, we present results from both analytical levels, consistent with prior studies (Nearing *et al.* 2022, Calgaro *et al.* 2023).

**Table 1:** Overview of analytical configurations evaluated: combinations of filtering, BH-based FDR correction, and taxonomic resolution (ASV and genus).

Configuration	Levels
Prevalence filtering	Yes/no
BH correction	Yes/no
Taxonomic level	ASV/genus

### 2.2 DAA methods

To investigate how signal detection varies between treatment groups (low versus severe toxicity), we applied several DAA methods. Each approach relies on specific statistical assumptions and offers unique advantages, together forming a comprehensive toolkit for assessing microbial biomarkers across treatment contrasts. These methods represent diverse statistical paradigms, including count-based [DESeq2 (Love *et al.* 2014)], compositional [ALDEx2 (Fernandes *et al.* 2014), ANCOM-BC (Lin and Peddada 2020a)], linear-model-based [LinDA (Zhou *et al.* 2022), LEfSe (Segata *et al.* 2011)], and zero-inflated [ZicoSeq (Yang and Chen 2022)] frameworks.

ALDEx2 models count data using a Dirichlet-multinomial distribution, followed by Monte Carlo sampling (K=128) and a centred log-ratio (CLR) transformation to account for compositionality. Differential abundance is then tested using the t-test. We used the original ALDEx2 package (aldex.clr function) (Fernandes *et al.* 2014) to ensure internal Benjamini-Hochberg (BH) correction, yielding adjusted *P-*value that control both FDR and type I error.

ANCOM-BC fits an offset-based log-linear model that corrects for sampling bias and compositional effects, providing bias-corrected estimates of log fold changes. We used the ANCOM-BC R package (v2.4.0) (Lin and Peddada 2020a) with manually tuned parameters. This model accounts for both compositionality and sparsity, improving interpretability relative to traditional ratio-based methods.

Initially developed for RNA-Seq data, DESeq2 (Love *et al.* 2014) has been widely adopted for microbiome analyses. It models raw counts using a negative binomial distribution, estimates size factors to normalize sequencing depth, and fits generalized linear models while applying a Wald test. The method inherently accommodates overdispersion and does not require prior data transformation. Both raw and BH-adjusted *P-*value were obtained from the final output.

LEfSe (Segata *et al.* 2011) combines non-parametric rank tests with LDA to identify features with consistent effects across classes. It first applies the Kruskal-Wallis test (and, optionally, Wilcoxon pairwise tests) before computing LDA scores that reflect discriminative effect size. Although widely used for its interpretability, LEfSe assumes independent observations and similarly distributed data, and it does not address zero inflation or compositional bias internally. To address this issue, a pseudo-count of 1e-06 was employed. Kruskal-Wallis *P-*value were extracted and BH-adjusted externally to ensure consistency across all DAA methods.

LinDA (Zhou *et al.* 2022) applies linear modelling to log-ratio–transformed counts, integrating bias correction and permutation-based variance estimation to improve robustness in compositional and heteroscedastic data. It assumes approximately normal residuals within the linear framework. The method provides both effect sizes (log fold changes) and FDR-adjusted significance estimates, enabling interpretation of differential abundance directionality.

ZicoSeq (Yang and Chen 2022) explicitly addresses zero inflation and compositionality by fitting a zero-inflated Gaussian mixture model. It transforms proportional data to proportions and applies a power transformation, with a square-root transformation applied by default. It derives permutation-based *P-*value that are subsequently adjusted with the BH procedure. This enhances statistical power while maintaining sensitivity in sparse microbiome data, which often contains many zero counts.

We compared the two extreme toxicity phenotypes: low (n=11) and severe (n=25). As no intermediate cases were available, all samples were retained. For each method (no-prevalence and prevalence filter), both raw and BH-adjusted *P-*value were reported to evaluate the stability of findings under different error-control regimes.

A comparative overview of the evaluated methods, including modelling assumptions, normalization strategies, and expected behaviour in small datasets, is provided in Table 2, available as supplementary data at *Bioinformatics Advances* online.

### 2.3 FDR control

In the context of the extensive hypothesis testing commonly performed in DAA, many methodologies employ FDR control techniques, most notably the BH procedure, with a significance threshold set at q=0.05. However, a q=0.1 was also evaluated (Benjamini and Hochberg 1995). This standardized procedure was uniformly applied across all six analytical methods, thereby enabling a comparative assessment of FDR-controlled outcomes derived from varied statistical models.

Furthermore, raw *P-*value are presented to underscore the sensitivity of these methods before any adjustments, which is especially pertinent in smaller cohorts where excessive corrections may obfuscate potentially meaningful signals. To strengthen biological interpretability, we also applied an additional filter of $|{\;\text{log}\;}_{2}\text{Fold}\;\text{Change}|>1$ (i.e. at least a two-fold change), allowing us to prioritize features demonstrating both statistical significance and meaningful effect sizes. While we recognize that the use of unadjusted *P-*value elevates the risk of false positives, their inclusion, alongside this fold-change threshold, serves a critical role in illustrating the trade-offs between methodological stringency and sensitivity across different approaches as suggested by Jiang *et al.* (2017).

### 2.4 Concordance evaluation

This study relies on a subset of the same real-world 16S V3-V4 dataset used in previous studies (Conde-Pérez *et al.* 2024a,b). Therefore, the findings here should be considered alongside earlier studies as a partial ground truth, a biological prior, or an expected reference signal. Nevertheless, concordance of bacterial features across analytical approaches was also considered an indirect indicator of robustness, as suggested by previous applications to non-simulated gut microbiome datasets (Wallen 2021) and previous benchmarking studies (Wallen 2021, Nearing *et al.* 2022, Yang and Chen 2022, Wirbel *et al.* 2024). To account for the limitations of small cohorts and the taxonomic resolution of the V3–V4 region, differential abundance analyses were conducted at the Genus and ASV levels. Concordant findings across methods and between taxonomic resolutions were prioritized, while discrepancies were documented to reflect method- or resolution-specific sensitivities.

### 2.5 Robustness and inter-method agreement analyses

To evaluate robustness in the absence of a gold-standard set of true positives, we examined the stability of the results under repeated subsampling using a stratified leave-k-out (LKO) resampling scheme as an internal validation procedure. All metrics reported in Section 3 are derived from this LKO procedure. At each iteration, a subset of samples was randomly omitted, and the entire analysis pipeline was executed again. Stratification ensured that the relative frequencies of the toxicity groups were maintained, thereby addressing class imbalance. Concretely, *k* samples were removed from each group at every iteration. Due to unequal group sizes and methodological requirements that impose a minimum sample size per group for model fitting (e.g. ZicoSeq), *k* was set to 1 for the low-toxicity group and 4 for the severe-toxicity group. This procedure was repeated for 100 iterations. The consistency of selected features, effect sizes, and directions of associations across iterations was used as a proxy for reproducibility and robustness to sample perturbation.

One of the agreement metrics used to quantify consensus between methods in the Results section is sensitivity, defined here as a measure of stability across folds. This metric corresponds to the detection rate, i.e. the proportion of LKO iterations (folds) in which a given taxon was deemed significant by a given method and multiple-testing correction scheme (noBH or BH). It is calculated as the mean of the logical variable significant (TRUE | FALSE) for each taxon across folds. The resulting values range from 0 to 1, where 0 indicates the taxon was never detected, values around 0.5 indicate detection in approximately half of the folds, and 1 indicates detection in all folds. This metric, therefore, quantifies the stability of a finding across resampled analyses rather than from a single run.

Furthermore, multiple complementary metrics were used to quantify the degree of agreement between the analytical methods. Feature overlap was assessed using the Jaccard index (Jaccard 1901), and results were summarized using the median and interquartile range (IQR), defined as the difference between the 75th and 25th percentiles. Agreement in effect size rankings was evaluated using Spearman’s and Kendall’s rank correlation coefficients (Spearman 1904, Kendall 1938), while directional agreement in estimated associations (low toxicity versus severe toxicity) was quantified using Cohen’s kappa statistic (Cohen 1960). Together, these measures provide a quantitative assessment of concordance in terms of feature selection, relative effect magnitude, and directionality across methods.

### 2.6 Reproducibility and software environment

The GitHub repository hosts the R Markdown (Rmd) scripts developed and employed in the present study. The repository also includes a data directory containing the phyloseq object corresponding to the original dataset analysed in this work, the data from the independent validation dataset, and the associated metadata for both datasets. All analyses were conducted in an R (v.5.4.0) environment using the RStudio integrated development environment (IDE), enabling full reproducibility of all figures and tables presented in the main manuscript and the Supplementary Materials, available as supplementary data at *Bioinformatics Advances* online. The GitHub repository for this project is available at https://github.com/elsamdea/DAA-CRC-Chemotoxicity-microbiome-analysis.

## 3 Results

This section presents the classification of microbial biomarkers into low- and severe-toxicity groups using several DAA methods, including ALDEx2, ANCOM-BC, DESeq2, LinDA, LEfSe, and ZicoSeq. A detailed analysis was conducted to evaluate the impact of prevalence filtering and multiple-testing correction. The dataset was analysed both with and without prevalence filtering, and in each scenario, results were evaluated with and without FDR correction using the BH procedure. Results are summarized by the number of detected biomarkers and their distribution across toxicity groups, as reported in Table 3, available as supplementary data at *Bioinformatics Advances* online. A summary of the characteristics, advantages, and limitations of the evaluated methodologies is provided in Table 2, available as supplementary data at *Bioinformatics Advances* online.

The results are presented in a structured sequence. First, analyses are performed on the study dataset (hereafter referred to as the primary dataset), initially focusing on the effect of the binary toxicity variable alone. Subsequently, models incorporating potential confounders (age, sex, and tumour location) are evaluated within the same dataset. These findings are then compared with results obtained from an independent external validation dataset. Finally, a dedicated subsection examines oral-associated bacterial taxa in both datasets to evaluate how consistently signals appear across biologically relevant toxicity groups.

### 3.1 Global structural analysis

**Figure 1:**
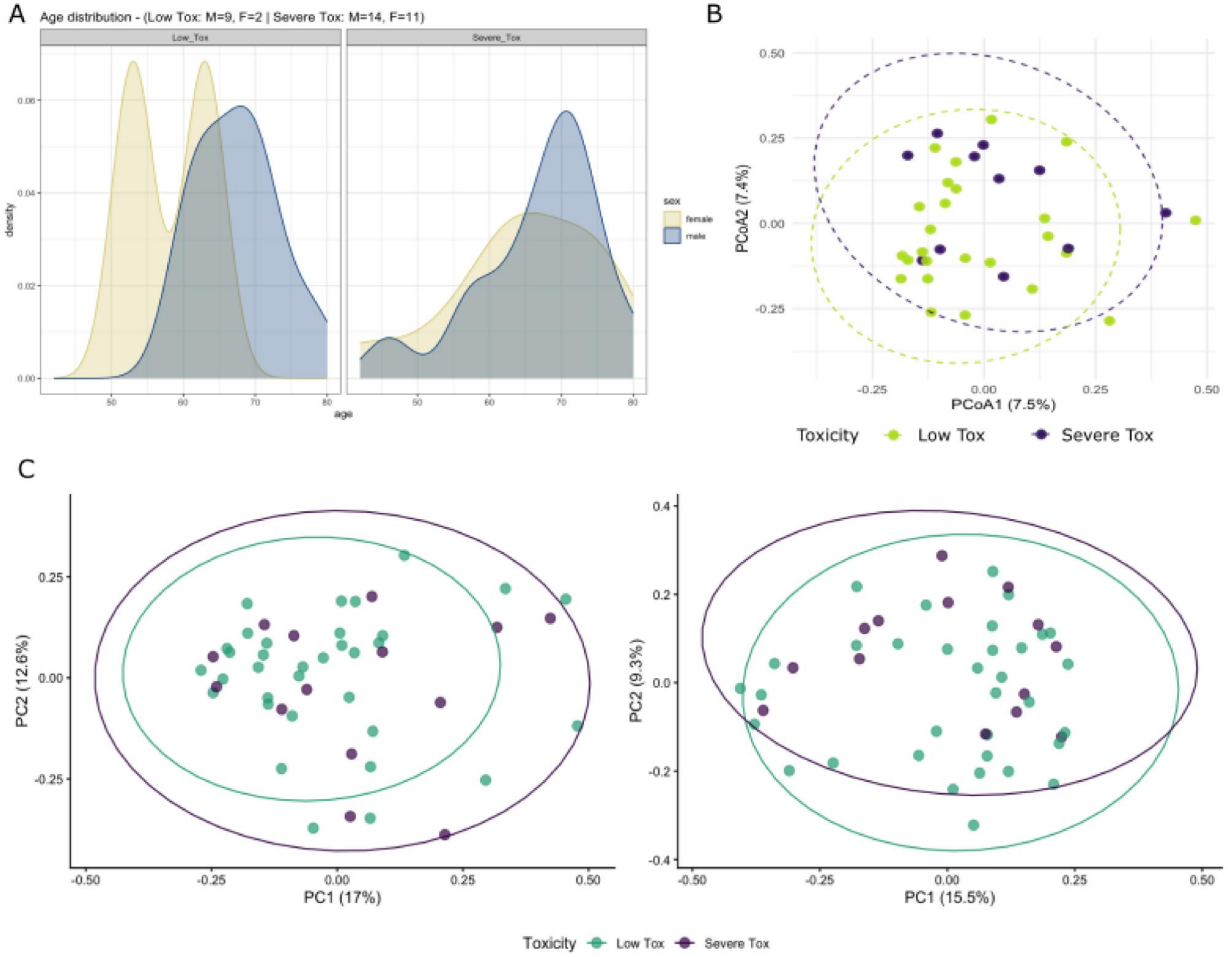
Global microbial community structure across toxicity groups. (A) Distribution of patients by toxicity group and sex in the primary dataset, showing the number of female and male patients within each toxicity category. (B) Principal Coordinates Analysis (PCoA) based on Bray–Curtis dissimilarity for the primary analysis cohort (*n* = 36), illustrating the high inter-individual variability between patients when comparing toxicity groups, with clusters largely overlapping. (C) PCoA of the external validation dataset (*n* = 48), based on Bray–Curtis dissimilarity (left) and Jaccard distance (right), also showing substantial overlap between toxicity groups, consistent with the high variability observed in the primary dataset.

We first examined global microbial community structure across toxicity groups to determine whether overall compositional differences were present. Figure 1 provides an overview of the dataset analysed in this study. Specifically, Panel 11A) illustrates the age distribution stratified by sex, showing that both male and female individuals were represented in each toxicity category within the 36-patient cohort of this study. A slight imbalance in female representation was observed between toxicity groups, with 2 females in the low-toxicity group and 11 in the severe-toxicity group. Panel 11B), a principal coordinates analysis (PCoA) based on Bray–Curtis dissimilarities, stratified by toxicity status, demonstrates substantial overlap among samples, with no evident clustering by toxicity group. This pattern is consistent with pronounced inter-individual heterogeneity, as commonly observed in human microbiome datasets. Additional detail is provided in Fig. F2, available as supplementary data at *Bioinformatics Advances* online, which displays additional multivariate characterization via five principal component analyses incorporating toxicity status (A), sequencing batch (B), sex (C), tumour location (D), and age distribution (E), none of which showed clear, dominant structuring of the dataset. Finally, Panel 11C) shows the distribution of samples across toxicity groups in the independent validation cohort (48 patients), comparing Bray-Curtis (left) and Jaccard (right) distance metrics, and similarly demonstrates extensive overlap between toxicity groups, in line with observations from the discovery cohort.

Permutational multivariate analysis of variance (PERMANOVA) corroborated these findings, indicating that toxicity status, sex, age group, and sequencing run did not significantly explain variation in overall microbial community composition (all P>.05). Although the sequencing run accounted for the largest proportion of variance ($R^{2}=14.8\%$), this effect was not statistically significant, suggesting limited batch effects. Consistently, the multipanel PCoA showed broad overlap among samples, with no clear clustering pattern by toxicity status or other covariates (i.e. sequencing batch, sex, tumour location, and age), indicating that global community structure is largely driven by inter-individual variability. Importantly, the absence of separation at the community level did not preclude the presence of reproducible taxon-specific differences, which were, therefore, examined in subsequent differential abundance and robustness analyses.

### 3.2 Detection stability and robustness across resampling iterations

To evaluate the robustness of differential abundance signals in the absence of a known ground truth, all analyses were performed within an LKO resampling framework. DAA analyses were repeatedly recomputed across subsampled datasets, and detection stability was quantified as the proportion of iterations in which a taxon was identified as differentially abundant.

**Figure 2:**
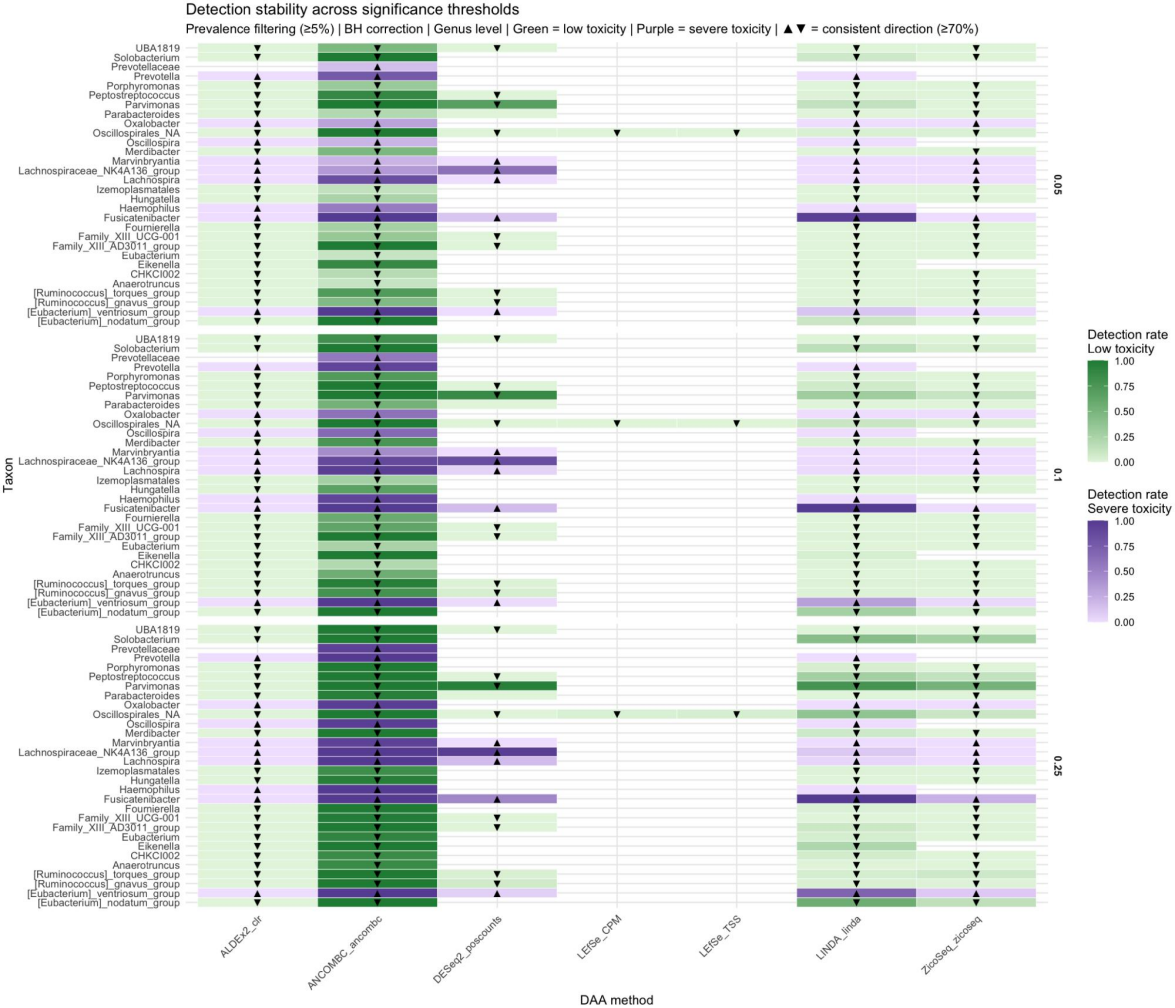
Detection stability across significance thresholds. Heatmap showing detection rates of differentially abundant taxa across differential abundance methods under prevalence filtering (≥5%) and BH correction at the genus level. Detection rates were estimated across resampling iterations for three significance thresholds (α = 0.05, 0.1, and 0.25). Green cells indicate taxa enriched in the low-toxicity group, whereas purple cells represent taxa enriched in the severe-toxicity group. Colour intensity reflects the frequency with which a taxon was detected across iterations. Triangular markers denote taxa for which the direction of association remained consistent across at least 70% of iterations. White cells indicate taxa not detected under any condition by the respective method; faint-coloured cells reflect a detection rate of zero but an assignable direction of association.

Detection frequencies were evaluated at three significance thresholds (α=0.05, 0.1, and 0.25) while concurrently monitoring the direction of enrichment between toxicity groups, as illustrated in Fig. 2. Genus-level results obtained under a prevalence threshold (≥5%) and BH multiple-testing correction are presented here, whereas the complete set of analytical configurations, encompassing ASV-level analyses and alternative filtering criteria, is displayed in Fig. F2, available as supplementary data at *Bioinformatics Advances* online. Marked heterogeneity in detection stability was observed across methods. ANCOM-BC identified the largest number of taxa with high detection frequencies across resampling iterations, followed in descending order by ALDEx2, LinDA, and ZicoSeq. In contrast, DESeq2 and LefSe detected fewer taxa overall and generally exhibited lower stability across iterations.

Despite these methodological discrepancies, only a relatively limited subset of taxa remained consistently detectable across analytical thresholds and resampling runs. In particular, taxa within the Lachnospiraceae family, such as *Fusicatenibacter* and *Lachnospira* or *Lachnospiraceae NK4A136 group*, together with *Parvimonas* and members of the Ruminococcaceae and *Eubacterium* groups, displayed comparatively stable detection and concordant effect direction across methods. The association between effect size and detection stability is depicted in Fig. F4, available as supplementary data at *Bioinformatics Advances* online. Taxa exhibiting both large effect sizes and high detection frequencies constituted the most compelling biomarker candidates, whereas multiple taxa with moderate effect sizes demonstrated low reproducibility across resampling iterations, indicating pronounced sensitivity to dataset composition and analytical framework. Collectively, these findings indicate that most differential abundance signals were method-dependent and unstable across resampling iterations. Nevertheless, a small subset of taxa remained reproducibly detectable across methods, significance thresholds, and analytical configurations, supporting their interpretation as the most robust biomarker candidates within this dataset.

### 3.3 Impact of prevalence filtering and multiple-testing correction

**Figure 3:**
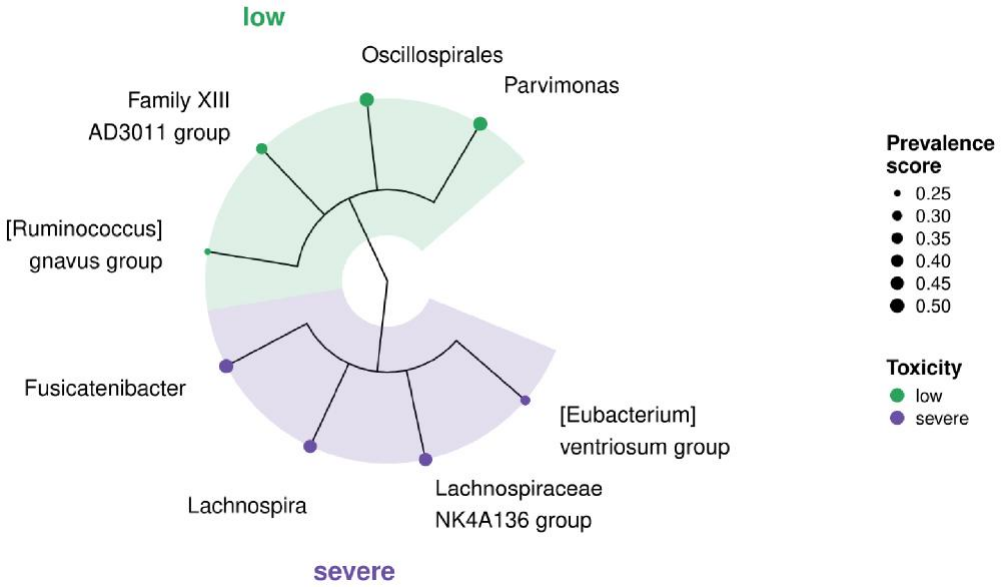
This cladogram compares chemotherapy-associated toxicity groups (low toxicity in green, severe toxicity in purple) based on the presence of four potential bacterial biomarkers. Each group exhibits a distinct bacterial signature and no biomarkers are shared between them.

Prevalence-based filtering and multiple-testing correction substantially influenced which taxa were identified as differentially abundant across all methods. Analyses conducted without prevalence filtering or FDR control generally yielded larger sets of putative differentially abundant taxa, whereas the combined application of both procedures produced smaller, more conservative feature sets. When prevalence filtering was applied, it disproportionately removed rare taxa (i.e. low-abundance and sparsely distributed taxa), thereby increasing concordance among methods and potentially enhancing biological interpretability, albeit at the cost of fewer statistically significant detections. Across methods, the joint implementation of prevalence filtering and FDR correction consistently resulted in smaller and more concordant sets of candidate taxa compared with unfiltered analyses. In some approaches, such as ALDEx2 and LEfSe, the combination of filtering and FDR control eliminated all significant features, as illustrated in Fig. 2 and the corresponding figure in the Supplementary Material (SF3), available as supplementary data at *Bioinformatics Advances* online. Despite these reductions, several taxa remained detectable across multiple analytical frameworks. In particular, genera such as *Fusicatenibacter*, *Lachnospira*, and *Parvimonas* were consistently identified across different modelling strategies, supporting their robustness to filtering and multiple testing correction, and suggesting that these taxa represent relatively robust signals within the dataset (Fig. 3).

### 3.4 Agreement and divergence across DAA methods

**Figure 4:**
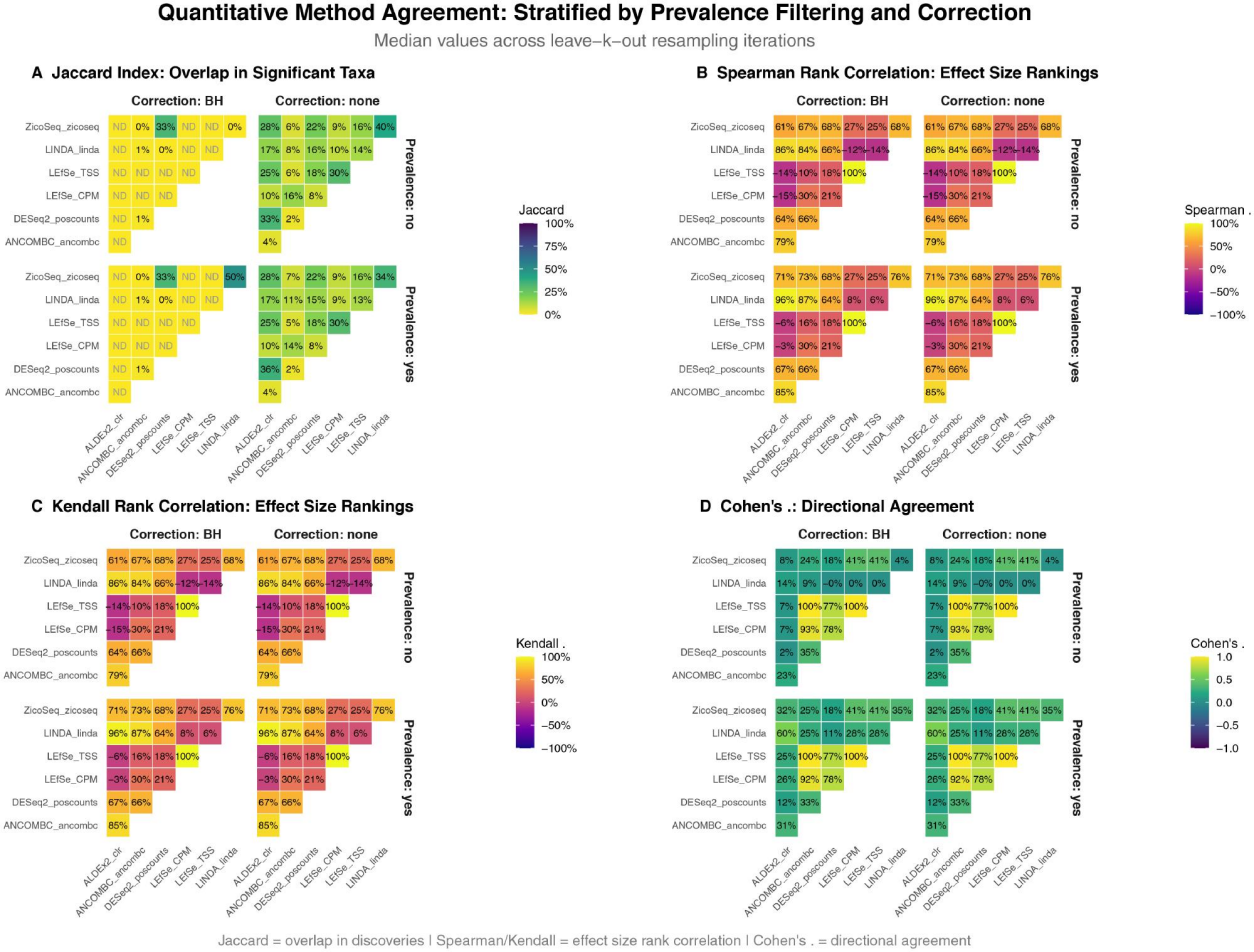
Quantification of agreement among differential abundance methods, considering taxa with and without prevalence thresholds of ≥5%, and using either BH multiple testing correction or no adjustment. Agreement is assessed using four complementary metrics: (A) overlap in significantly detected taxa, quantified by the Jaccard index; (B) concordance in the ranking of effect sizes, quantified by Spearman’s (ρ), more sensitive to differences at the extremes of the ranking; (C) concordance in the ranking of effect sizes, quantified by Kendall’s (τ), more robust to outliers and tied values; and (D) concordance in the direction of effects, quantified by Cohen’s κ. Reported values correspond to the median across leave-*k*-out resampling iterations. ND indicates that the metric could not be estimated for this stratum: no significant taxa were detected in either method (Jaccard); insufficient reliable feature pairs (n≥10%) were available (Spearman, Kendall); or insufficient taxa with directional data were present (Cohen’s κ).

Agreement between DAA methods was evaluated using four complementary metrics: taxonomic overlap (Jaccard index), rank concordance of effect sizes (Spearman’s ρ and Kendall’s τ), and agreement in the direction of effects (Cohen’s κ), as shown in Fig. 4. Unless otherwise stated, analyses were restricted to taxa with prevalence ≥5% and adjusted using BH multiple testing correction.

Based on the Jaccard index, taxonomic overlap was generally low (see Fig. 4a), with most pairwise Jaccard values below 40% and many comparisons yielding values close to zero or not defined (ND) after BH correction. Slightly higher overlap was observed in the uncorrected analyses, particularly between LinDA, and ZicoSeq and, to a lesser extent, DESeq2. In contrast, ANCOM-BC and LEfSe generally exhibited minimal overlap with the other methods, indicating that they tend to identify largely distinct sets of taxa. As expected, the two LEfSe normalization strategies (CPM and TSS) showed perfect overlap across all conditions.

Despite the limited overlap in detected taxa, several methods showed substantial concordance in effect-size rankings, as observed in Fig. 4b (Spearman’s ρ) and C (Kendall’s τ). ALDEx2, ANCOM-BC, LinDA, and ZicoSeq consistently displayed strong positive correlations in both metrics when all tested taxa were considered (i.e. prior to filtering based on BH-adjusted significance), while DESeq2 showed moderate agreement with these approaches. By contrast, comparisons involving LEfSe were often weak, negative, or not estimable (ND), consistent with its limited overlap with other methods, although, as expected, the two LEfSe normalization strategies showed perfect rank concordance. Agreement in the direction of effects (i.e. association with lower versus higher toxicity) was assessed using Cohen’s κ (Fig. 4d). Near-perfect directional agreement was largely restricted to the two LEfSe normalization approaches, whereas other method pairs showed only low to moderate κ values. Comparisons of ANCOM-BC and DESeq2 with LEfSe also exhibited moderate to high directional agreement in certain scenarios, although this trend was less uniform across conditions. Notably, several method pairs with strong rank concordance, including LinDA and ZicoSeq, displayed limited agreement in effect direction, indicating that similar effect-size rankings do not necessarily translate into consistent biological interpretation.

Overall, these results indicate that LinDA, ZicoSeq, ALDEx2, and ANCOM-BC produced the strongest agreement in effect-size rankings, whereas LEfSE consistently exhibited weaker concordance with other approaches. ANCOM-BC showed high detection sensitivity but relatively limited overlap with other methods, suggesting that increased sensitivity may come at the cost of lower inter-method reproducibility. More broadly, agreement between DAA methods strongly depended on the metric considered: methods may show substantial concordance in effect-size rankings while still differing considerably in the specific taxa detected and the inferred direction of differential abundance. Equivalent analyses for the primary dataset, including covariates, and for the external validation dataset are provided in Figs F7 and F11, available as supplementary data at *Bioinformatics Advances* online, respectively. While the inclusion of covariates resulted in generally weaker concordance and a higher proportion of non-estimable comparisons, the external validation dataset showed more consistent agreement across methods, with stronger rank concordance and improved directional agreement.

### 3.5 Confounder-adjusted models

An additional layer of analysis incorporated confounders (age, sex, and tumour location) into the DAA models, which support multivariable inference (ALDEx2, ANCOM-BC, LinDA, and ZicoSeq) to be evaluated by LKO for adjusted inference. The age of participants was categorized into three groups: under 60, 60–69, and 70 and older. Tumour location was categorized as right, left, transverse colon, and rectum. Sex was categorized as binary (female versus male). Equivalent analyses are summarized in Figs F6 and F7, available as supplementary data at *Bioinformatics Advances* online. Overall, the inclusion of covariates reduced the number of significant taxa and weakened inter-method concordance, particularly after BH correction. Nevertheless, several of the main patterns observed in primary analysis were preserved. In particular, *Fusicatenibacter*, *Parvimonas*, and members of Lachnospiraceae and Ruminoccocaceae family remained consistently detected across multiple methods, whereas ANCOM-BC continued to identify the largest number of differentially abundant taxa under prevalence-filtered conditions. Agreement analyses showed lower overlap and directional concordance compared with the primary dataset, although LinDA, ZicoSeq, ALDEx2, and ANCOM-BC still exhibited the strongest consistency in effect-size rankings.

### 3.6 External validation dataset

Analyses performed in the external validation dataset are summarized in Figs F10 and F11, available as supplementary data at *Bioinformatics Advances* online. Similar to the primary dataset, substantial microbiome heterogeneity was observed across toxicity groups, with no clear separation in ordination analyses. Despite this variability, several methodological patterns were reproduced. ANCOM-BC again showed the highest detection sensitivity across significance thresholds, whereas agreement in effect-size rankings remained strongest among LinDA, ZicoSeq, ALDEx2, and ANCOM-BC. Compared with the original dataset, the validation cohort showed improved directional agreement and stronger rank concordance across methods. At the taxonomic level, several genera identified in the primary analysis, including *Fusicatenibacter* and members of the Oscillospirales order, remained consistently associated with toxicity groups, although some taxa displayed inconsistent directional assignments across methods.

### 3.7 Oral-associated bacteria

Given the accumulating evidence implicating the oral microbiota in CRC, we conducted a focused analysis of oral-associated bacterial genera (Figs F5, F8, and F12, available as supplementary data at *Bioinformatics Advances* online). Following LKO resampling as previously described, the primary dataset identified *Porphyromonas* as the most consistently detected oral-associated genus across all analytical methods (Fig. F5, available as supplementary data at *Bioinformatics Advances* online). *Peptostreptococcus*, *Parvimonas*, *Fusobacterium*, and *Actinomyces* were also frequently detected, although their directions of association varied across methods in some instances. Across all methods, ANCOM-BC exhibited the highest detection frequencies for oral-associated taxa.

Despite generally low detection frequencies, several oral genera demonstrated consistent directional patterns in covariates adjustment (Fig. F8, available as supplementary data at *Bioinformatics Advances* online). *Dialister* and *Mogibacterium* were predominantly associated with severe toxicity across methods, whereas *Parvimonas* and *Fusobacterium* were more frequently associated with low toxicity. Nonetheless, discordant classifications were observed for several taxa, particularly in LEfSe and DESeq2 analyses.

In the external validation dataset, *Parvimonas*, *Prevotella*, and *Veillonella* were consistently associated with low toxicity across methods, whereas *Actinomyces*, *Dialister*, and *Mogibacterium* were repeatedly associated with severe toxicity (Fig. F12, available as supplementary data at *Bioinformatics Advances* online). Although some genera exhibited inconsistent directional assignments between methods, the overall patterns supported the reproducibility of a subset of oral-associated taxa across independent datasets.

## 4 Discussion

In this study, we evaluated DAA methodologies to determine how methodological variability influences biomarker detection in patients with CRC experiencing chemotherapy-related toxicity. Rather than comparing healthy and diseased individuals, our analyses focused on clinically heterogeneous patient subgroups, a setting expected to increase microbiome variability and reduce signal reproducibility across analytical frameworks.

Our results demonstrate clear differences in performance among methods. ANCOM-BC showed the highest detection sensitivity and stability across analytical configurations, including prevalence filtering, covariate-adjusted analyses, and external validation. LinDA and ZicoSeq displayed similar behaviour, with strong concordance in effect-size rankings and comparatively robust performance under compositional and sparse microbiome conditions. In contrast, ALDEx2 exhibited conservative behaviour with reduced sensitivity after BH correction under conditions of high intra-group variability and small sample sizes, consistent with previous reports (Cappellato *et al.* 2022, Pelto *et al.* 2025). LEfSe failed to retain significant taxa under stringent filtering conditions, due to its lack of internal multiple-testing correction (Segata *et al.* 2011, Nearing *et al.* 2022, Khleborodova *et al.* 2024), and consistently showed weaker agreement with other methods. DESeq2 remained sensitive for taxa detection but showed greater variability depending on normalization and dispersion settings.

A central finding of this study was the discrepancy between overlap in statistical significance and concordance in effect-size estimates across methods. Taxonomic overlap, quantified using the Jaccard index, remained low, particularly following BH correction and prevalence filtering, indicating that different methods frequently identified largely disjoint sets of statistically significant taxa. Nevertheless, several methods, especially ANCOM-BC, LinDA, ZicoSeq, and ALDEx2, exhibited strong concordance in effect-size rankings despite the limited overlap in their statistically significant discoveries. This pattern suggests that methodological divergence arises predominantly from differences in statistical thresholding rather than from fundamentally incompatible underlying abundance structures. In contrast, agreement in the direction of differential abundance was substantially lower, implying that similar ranking patterns do not necessarily translate into consistent biological interpretations.

In this study, a pragmatic framework for method selection is proposed that is explicitly grounded in key dataset characteristics, including sparsity, compositionality, sample size, zero inflation, and covariate structure. The methodological comparison (Table 2, available as supplementary data at *Bioinformatics Advances* online), together with the complementary practical guidelines provided in the Supplementary Material, available as supplementary data at *Bioinformatics Advances* online, offers a systematic basis for context-dependent analytical decisions that is consistent with our empirical results and prior benchmarking efforts. Methods that explicitly account for compositionality, such as ANCOM-BC, LinDA, and ZicoSeq, generally exhibited superior performance in sparse and/or compositional datasets. ANCOM-BC demonstrated stable performance across a wide range of scenarios; LinDA afforded greater flexibility for covariate-adjusted analyses; and ZicoSeq was robust to zero inflation and outliers, although it required comparatively larger sample sizes (e.g. ≥10 samples per group) to yield reliable performance. For small-sample datasets, ALDEx2 provided conservative inference that may reduce false-positive findings at the cost of reduced statistical power (Weiss *et al.* 2017, Swift *et al.* 2023). In contrast, DESeq2 remained relatively sensitive but necessitated cautious interpretation in compositional contexts. Finally, LEfSe can be valuable for exploratory biomarker discovery and effect-size visualization, but its lack of explicit FDR control may inflate false-positive rates, particularly in small or heterogeneous cohorts. In conclusion, although ANCOM-BC exhibited the most consistently stable performance across conditions, method selection should remain context-specific and be informed by dataset characteristics and analytical priorities, rather than relying on a single, universally applicable analytical framework.

Despite considerable methodological variability, only a limited subset of taxa remained reproducibly detectable across resampling analyses, analytical configurations, and external validation. Among these, *Parvimonas*, *Fusicatenibacter*, and members of the Lachnospiraceae family emerged as the most stable signals. *Parvimonas* was consistently detected across multiple methods and analyses, in agreement with previous reports linking this genus to CRC-associated microbial signatures (Conde-Pérez *et al.* 2024a,b). Interestingly, although *Parvimonas* has frequently been associated with poor CRC prognosis and immunosuppressive tumour environments (Löwenmark, Löfgren-Burström, *et al.* 2022, Löwenmark, Köhn, *et al.* 2024), in our cohort it was more frequently associated with lower toxicity, suggesting a context-dependent role in treatment-associated microbiome dynamics.

Similarly, *Fusicatenibacter* was consistently associated with severe toxicity across all analytical approaches and validation procedures. This observation aligns with previous reports linking this genus to chemotherapy-induced diarrhoea and haematological toxicity (Kawasaki *et al.* 2023, Xiaofeng *et al.* 2023), thereby reinforcing its putative role as a toxicity-associated taxon. In addition, members of the family Lachnospiraceae, including *Lachnospira* and the *Lachnospiraceae NK4A136 group*, were repeatedly associated with severe toxicity, despite their frequently reported beneficial metabolic functions (Zhao *et al.* 2023). Conversely, taxa belonging to the Ruminococcaceae family, including the *Ruminococcus torques* and *Ruminococcus gnavus* groups, have been associated with lower toxicity and have previously been linked to improved treatment outcomes and immune resilience (Lee *et al.* 2021). This apparent contradiction suggests that the functional role of bacteria that produce short-chain fatty acids (SCFAs) may shift under treatment-induced stress or depend on ecological context and host–microbiome interactions. Collectively, these results highlight how strongly microbiome–treatment interactions depend on context and reflect substantial ecological complexity (Zhao *et al.* 2023, Böhm *et al.* 2025, Hillege *et al.* 2025, Piccinno *et al.* 2025).

Robustness analyses supported the stability of several microbial associations. Covariate-adjusted models including age, sex, and tumour location consistently retained taxa such as *Parvimonas* and *Fusicatenibacter*, suggesting these relationships were not driven by major confounders. Ageing is a key variable due to its links to pro-inflammatory immune shifts, reduced abundance of SCFAs-producing bacteria, and immunosenescence (Pandey *et al.* 2023). Sex shapes the gut microbiota via hormonal and immune mechanisms, including microbial regulation of oestrogen metabolism, and is a major covariate influencing treatment response in ovarian cancer (Zhang *et al.* 2025). In CRC, sex-related differences have been associated with tumour distribution and hormone-dependent signalling (Wu *et al.* 2024). Tumour location defines distinct intestinal niches that structure microbial communities and modulate therapeutic outcomes (Shi *et al.* 2020, Pandey *et al.* 2023, Böhm *et al.* 2025). More broadly, host–microbiome interactions can alter drug pharmacokinetics and pharmacodynamics, contributing to inter-individual variability in chemotherapy efficacy and toxicity (Böhm *et al.* 2025, Hillege *et al.* 2025). Rigorous adjustment for these variables is, therefore, essential to avoid spurious associations driven by medications, diet, and lifestyle. Large-scale benchmarking of real-world cohorts shows that inadequate control of confounders, including medication use, can yield misleading or artefactual microbial associations (Wirbel *et al.* 2024). While covariate adjustment enhances interpretability by reducing spurious associations, residual confounding cannot be fully excluded. Unmeasured or incompletely captured factors, such as dietary patterns, concomitant medications, and lifestyle, may still influence microbiome composition and treatment outcomes, thereby contributing to heterogeneity across studies (Wirbel *et al.* 2024, Böhm *et al.* 2025, Hillege *et al.* 2025).

Likewise, partial concordance between the primary dataset and an independent validation cohort, despite divergent sequencing strategies, suggests that a subset of microbial signals may be robust to variation in analytical pipelines and sequencing platforms (Knight *et al.* 2018). Analyses of α and β diversity in the validation dataset similarly demonstrated considerable within-group heterogeneity across toxicity strata, without evidence of distinct clustering or separation in ordination space. This pattern is consistent with the heterogeneous microbial architecture observed in the primary cohort and underscores the inherent difficulty of identifying reproducible biomarkers in clinical microbiome studies when comparing responders versus non-responders, particularly in small pilot cohorts that require independent validation (Shi *et al.* 2020, Bukavina *et al.* 2024). Notably, the distribution of toxicity cases differed between the primary and validation cohorts, with a higher proportion of severe toxicity cases in our dataset and a greater representation of low-toxicity patients in the validation cohort. Such imbalances in group composition may affect statistical power and detection rates across differential abundance methods, particularly in small microbiome cohorts.

Several limitations should be considered. The study relied primarily on a relatively small and clinically heterogeneous cohort, which may reduce statistical power and limit generalizability. In addition, microbiome benchmarking studies lack a known biological ground truth, complicating direct evaluation of method accuracy (Nearing *et al*. 2022, Wirbel *et al.* 2024, Pelto *et al.* 2025). Variability introduced by sequencing platforms, preprocessing pipelines, and analytical frameworks may further contribute to discrepancies between studies and limit reproducibility across cohorts.

These findings collectively suggest that the choice of analytical methodology exerted a substantial impact on microbiome biomarker detection within clinically heterogeneous cohorts. Compositionally informed approaches, including ANCOM-BC, LinDA, and ZicoSeq, exhibited the most consistently robust performance across diverse analytical scenarios, whereas dependence on a single statistical framework frequently produced method-specific signatures. Across resampling analyses and independent validation, only a limited subset of taxa, including *Fusicatenibacter*, *Lachnospira*, and *Parvimonas*, was consistently detected. This observation aligns with previous longitudinal microbiome studies in oncology, which show that treatment-related microbiome changes often involve relatively small subsets of taxa against a background of high inter-individual variability and overall community stability (Faith *et al.* 2013, Wallen, 2021, Nearing *et al.* 2022, Hillege *et al.* 2025). More broadly, these results advocate implementing multi-method consensus strategies and resampling-based validation procedures to enhance the reproducibility and biological interpretability of microbiome differential abundance analyses. Future studies incorporating larger cohorts, longitudinal sampling, and complementary sequencing technologies will be essential to validate these observations and clarify the role of the gut microbiota in modulating chemotherapy-related toxicity.

## Supplementary Material

vbag148_Supplementary_Data

## Data Availability

The data supporting this study are publicly available in the National Centre for Biotechnology Information (NCBI) Sequence Read Archive (SRA) database under the accession codes PRJNA911189 and PRJNA893853. The complete metadata set used in this paper is available in our GitHub repository (see Section 2.6). The external validation dataset used in this study will be available in the GitHub repository for this paper (see Section 2.6). At the same time, it can be downloaded directly from the original paper (Hillege *et al.* 2025).
