# Supplementary material for: Microbiome differential abundance methodologies to detect relevant taxa associated with chemotherapy toxicity rate in colorectal cancer
Source: Bioinform Adv. 2026 Jun 24;6(1):vbag148. doi: 10.1093/bioadv/vbag148 (PMC13317941; doi:10.1093/bioadv/vbag148)
Supplement: vbag148_Supplementary_Data [file vbag148_supplementary_data.pdf]

Contents

1 Methods **1**

1.1 Treatment Summary Table 2

1.2 DAA methodologies Summary Table 3

1.3 Example of a Single Iteration Evaluating DAA Approaches 6

2 Supplementary Figures **8**

2.1 Supplementary Figures (Primary dataset) 8

2.2 Supplementary Figures (Confounder-adjusted models) 13

2.3 Supplementary Figures (Validation Dataset) 16

Methods

The following table describes the characteristics of the cohort studied in this research and the main variables selected by the oncologists to develop the study's primary target variable, toxicity.

Treatment Summary Table

Table 1. Treatment Summary Table. Detailed treatment and toxicity variables (Supplementary Table S1)

Variable	Level	Low (n = 11)	Severe (n = 25)
QTP	1 (5-FU only)	1 (9%)	6 (24%)
	2 (5-FU+OX, 3 mo)	6 (55%)	11 (44%)
	3 (5-FU+OX, 6 mo)	4 (36%)	8 (32%)
RED_FP ≥20%	0 (none)	11 (100%)	3 (12%)
	1 (80%)	—	15 (60%)
	2 (60%)	—	2 (8%)
	3 (suspension)	—	5 (20%)
RED_OXALI ≥20%	0 (none)	9 (82%)	2 (8%)
	1 (80%)	—	10 (40%)
	2 (60%)	2 (18%)	4 (16%)
	3 (suspension)	—	9 (36%)
RTP	0 (no)	10 (91%)	23 (92%)
	1 (yes)	1 (9%)	2 (8%)
Emesis grade	0–1	10 (91%)	18 (72%)
	2	1 (9%)	7 (28%)
	≥3	—	—
Diarrhoea grade	0	8 (73%)	7 (28%)
	1	2 (18%)	12 (48%)
	2	1 (9%)	4 (16%)
	3	—	2 (8%)
EPP grade	0	11 (100%)	21 (84%)
	1–2	—	3 (12%)
	3	—	1 (4%)
	4–5	—	—
Neuropathy grade	0	3 (27%)	7 (28%)
	1	7 (64%)	15 (60%)
	2	1 (9%)	3 (12%)
Thrombocytopenia grade	0	11 (100%)	14 (56%)
	1	—	7 (28%)
	2	—	2 (8%)
	3	—	2 (8%)
Neutropenia grade	0	8 (73%)	6 (24%)
	1	2 (18%)	10 (40%)
	2	1 (9%)	5 (20%)
	3	—	3 (12%)
	4	—	1 (4%)

Dose-reduction coding: 0 = none, 1 = 80 %, 2 = 60 %, 3 = suspension. Symptom grades are per CTCAE v5.0 (0-5). Abbreviations: Erythropoietic Protoporphyria (EPP). Full data dictionary is available with the public dataset (PRJNA911189, PRJNA893853).

DAA methodologies Summary Table

As outlined in the main manuscript, Table S2 describes the six DAA methodologies. The final column focuses specifically on the observations made in the small dataset under review and the findings of previous studies in this field.

Table 2. Please find below a summary of the DAA methodologies, including a review of the model principle, the input type, normalisation, year, advantages and limitations, as well as an explanation of how it works in low sample size datasets. The main manuscripts refer to this table as Table S2.

Method	Model principle	Input	Normalisation	Year	Key advantages	Key limitations	Small-n notes
ALDEx2[1]	Dirichlet–MC sampling	Raw counts	CLR (MC)	2014	Robust to compositionality; low FP	Conservative; lower power; less sensitive	Stable via MC sam pling, but no between-group differences
ANCOM-BC[2]	Log-linear approach with sample-specific offsets (bias correction)	Raw counts	Log + bias correction	2020	Corrects sampling-fraction bias; FDR control	Power drops for rare taxa; unstable at very small n	Unstable if n is tiny
DESeq2[3]	NB-GLM with size factors	Raw counts	Size factors	2014	Mature; high sensitivity	Inflated FDR on compositional data; sparsity-sensitive	Works with shrinkage; careful dispersion
LEfSe[4]	KW + Wilcoxon with LDA (effects)	RA	TSS / CPM	2011	Simple; intuitive outputs	No FDR control; No compositionality; Norm.dependent	Prone to FP in small n, fails when differences are mainly within-group
LinDA[5]	Linear model on CLR with bias correction	Counts or Prop.	CLR + bias correction	2022	Balanced FDR/power; supports complex designs	Assumes log-normal; zero handling	Good if n is modest; power loss when n is very small
ZicoSeq[6]	Reference-based log-ratio (beta-mixture) testing with winsorization	Counts or RA	Log-ratio aware	2022	Robust to zeros/ outliers; composition-aware	Computationally heavier than others	Power loss when detecting rare taxa in small groups.

Abbrev.: Prop = Proportions; RA = Relative Abundances; TSS = total-sum scaling; CPM = Counts Per Million; CLR = Centred Log-Ratio; NB-GLM = Negative Binomial GLM; FP = False Positives; FDR = False Discovery Rate; Norm. = Normalisation. Note: DESeq2[3], size factors normalisations refers to medianofratios, go to (3)for further information.Superscripts [1]–[6]: ALDEx2[1] (1); ANCOM-BC[2] (2); DESeq2[3] (3); Segata (4); LinDA[5] (6); ZicoSeq[6] (5).

Practical guidelines for method selection. Based on both our empirical results and previous benchmarking studies, we provide the following recommendations:

- **High sparsity / zero inflation:** ANCOM-BC, ZicoSeq.
- **Strong compositional constraints:** ANCOM-BC, LinDA.
- **Small sample size:** ALDEx2 (conservative), ANCOM-BC (with caution).
- **Presence of covariates:** ANCOM-BC, LinDA, ZicoSeq.
- **Exploratory biomarker discovery:** LEfSe.
- **Larger datasets / higher sensitivity:** ANCOM-BC, LinDA, ZicoSeq, DESeq2 (with careful normalization).

Example of a Single Iteration Evaluating DAA Approaches

Table 3. Example of one iteration evaluating the four approaches: prevalence filtering (no vs. yes), correction (none vs BH), also separated by ASVs (A) and Genus (G) taxa. A range of significant and abundant biomarkers were identified using the DAA methods, evaluated both with and without a prevalence filter and Benjamini-Hochberg (BH) *p*-value correction. Significance thresholds were defined as follows: unfilt, noBH: $p < 0.05$ and $|\mathrm{LFC}| > 1$; unfilt, BH: adjusted p (or q) < 0.10 and $|\mathrm{LFC}| > 1$; filt, noBH: $p < 0.05$ and $|\mathrm{LFC}| > 1$; filt, BH: adjusted $p < 0.05$ and $|\mathrm{LFC}| > 1$. Abbreviations: LECPM = LEfSe-CPM; LETSS = LEfSe-TSS; LFC = Log Fold Change; (TB) = total biomarkers; (LT) = low-toxicity class; (ST) = severe-toxicity class; (NoBH) = uncorrected *p*-value; (BH) = Benjamini-Hochberg correction.

		ALDEx2			ANCOM-BC			DeSeq2			LECPM			LETSS			LinDA			ZicoSeq		
		TB	LT	ST	TB	LT	ST	TB	LT	ST	TB	LT	ST	TB	LT	ST	TB	LT	ST	TB	LT	ST
UnFilt	NoBH(G)	11	7	4	13	7	6	7	2	5	9	4	5	9	4	5	4	2	2	1	1	0
	NoBH(A)	12	5	7	18	9	9	6	0	6	13	8	5	13	8	5	12	11	1	18	10	8
	BH(G)	0	0	0	1	0	1	2	1	1	0	0	0	0	0	0	1	0	1	1	1	0
	BH(A)	0	0	0	1	0	1	1	0	1	0	0	0	0	0	0	1	0	1	1	1	0
Filt	NoBH(G)	5	0	5	15	11	4	1	0	1	9	5	4	9	5	4	18	14	4	1	1	0
	NoBH(A)	5	0	5	21	13	8	1	0	1	11	3	8	9	5	4	54	43	11	23	12	11
	BH(G)	0	0	0	4	3	1	1	0	1	0	0	0	0	0	0	2	1	1	1	1	0
	BH(A)	0	0	0	2	0	2	1	0	1	0	0	0	0	0	0	12	11	1	0	0	0

Supplementary Figures

Supplementary Figures (Primary dataset)

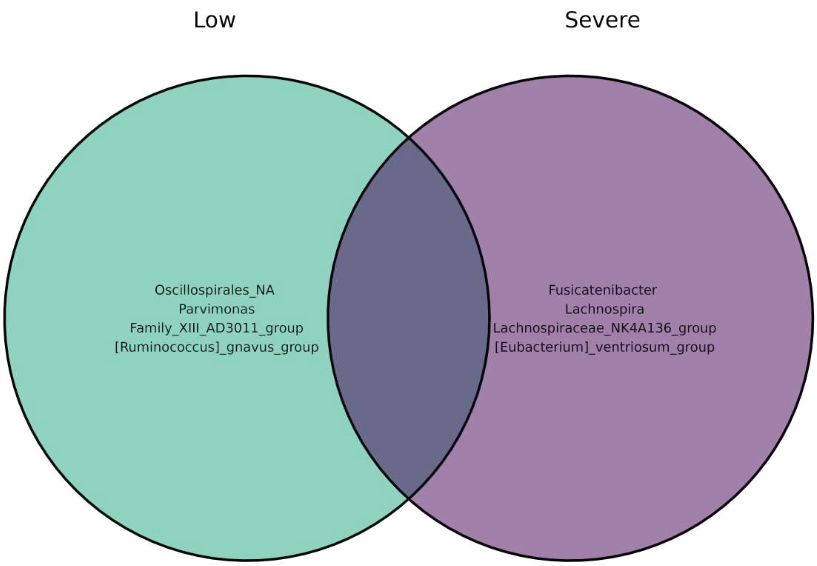

Fig. 1. Venn diagram of the primary dataset. This Venn diagram compares chemotherapy-associated toxicity groups based on the presence of four potential bacterial biomarkers. Each group exhibits a distinct bacterial signature, and no biomarkers are shared between them. Low toxicity is indicated in green, and severe toxicity in purple. (Referenced in manuscript as Supplementary Figure F1).

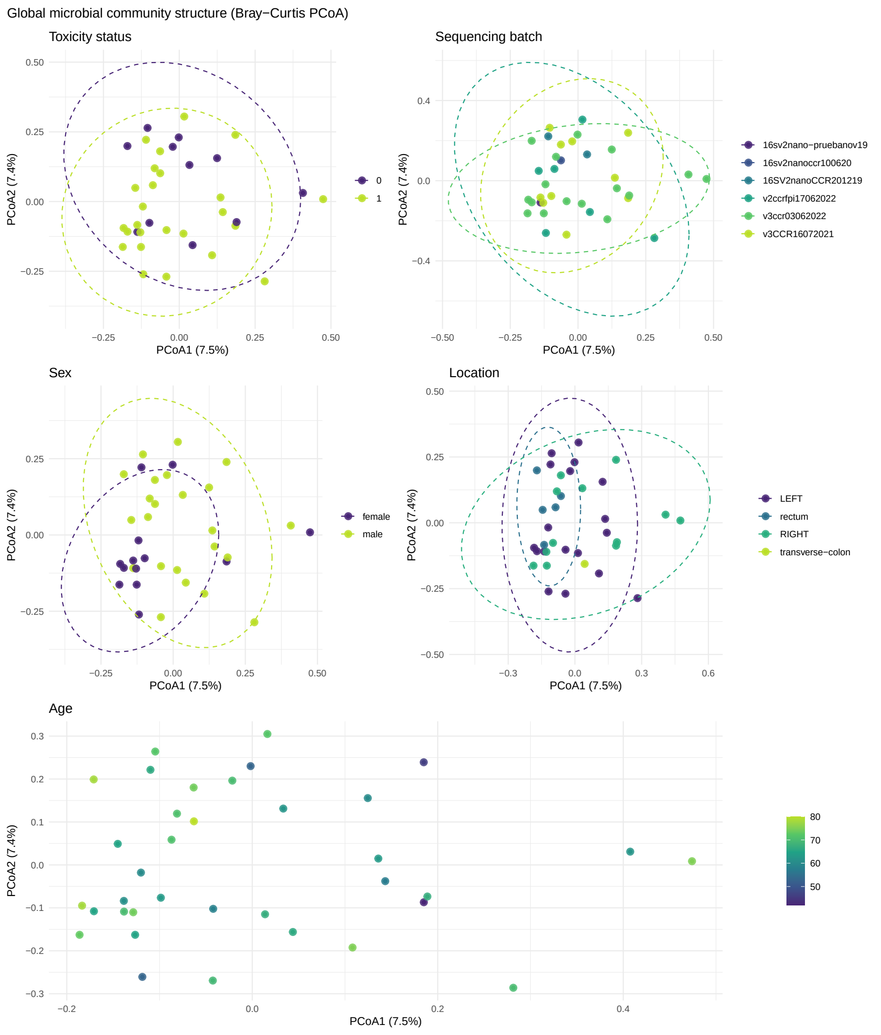

Fig. 2. Principal Coordinates Analysis (PCoA) of the primary dataset showing global patterns of sample similarity using Bray–Curtis dissimilarity. Each point represents a sample and is coloured according to (A) toxicity status, (B) sequencing batch, (C) sex, (D) tumour location, and (E) age. This dimensionality reduction provides an overview of sample distribution in multivariate space and allows visual assessment of clustering patterns and potential confounding variation not captured by recorded covariates. (Referenced in manuscript as Supplementary Figure SF2).

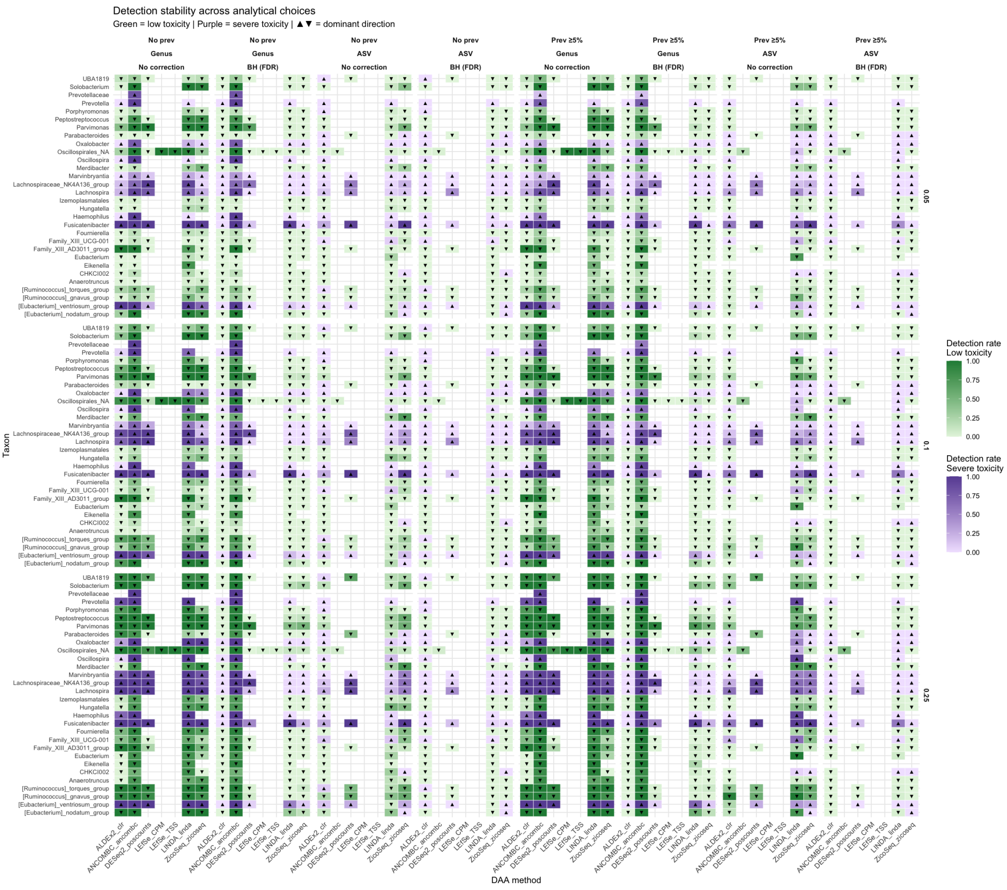

Fig. 3. Multi-approach heatmap from primary dataset. Heatmap summarising the detection of the top 30 bacterial taxa across multiple differential abundance analysis (DAA) methods under different analytical settings: two prevalence filtering strategies (no filtering vs. 5% filtering) and two multiple-testing correction approaches (none vs. Benjamini–Hochberg (BH) correction). Analyses were performed at both genus and ASV levels, a commonly recommended strategy in microbiome studies. Three significance thresholds (q-values) are shown on the right side of the plot to illustrate how detection varies across methods and thresholds. Rows represent the selected oral taxa, and columns represent the DAA methods. Cell colours indicate the detection rate (0–1), defined as the proportion of analyses in which a given taxon was identified as differentially abundant by each method. Taxa are additionally annotated according to the toxicity group in which they were enriched, as classified by the respective DAA methods. White cells indicate taxa not detected under any condition by the respective method; faint-coloured cells reflect a detection rate of zero but an assignable direction of association. (Referenced in manuscript as Supplementary Figure SF3).

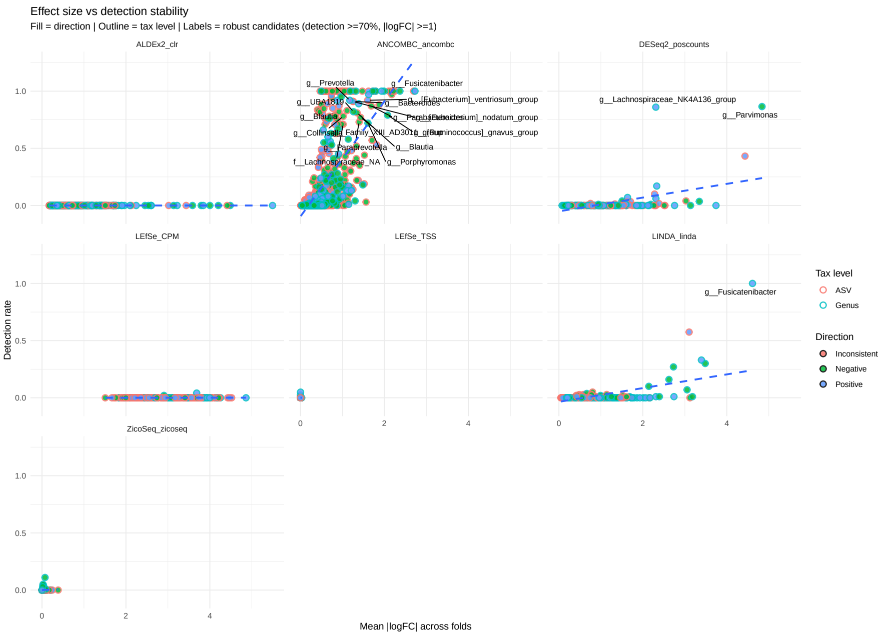

Fig. 4. Effect size versus detection stability across differential abundance methods. Scatter plots showing the relationship between mean absolute log-fold change (—logFC—) and detection rate across resampling iterations for each differential abundance method. Each point represents a taxon identified at either the ASV or genus level. Detection rate indicates the proportion of iterations in which the taxon was detected as differentially abundant. Point fill color represents the direction of association (positive or negative effect), while the outline indicates taxonomic level (ASV or genus). Labels highlight candidate taxa exhibiting both high detection stability (70% of iterations) and large effect sizes (—logFC— ≥ 1), representing the most robust biomarker candidates. (Referenced in manuscript as Supplementary Figure SF4).

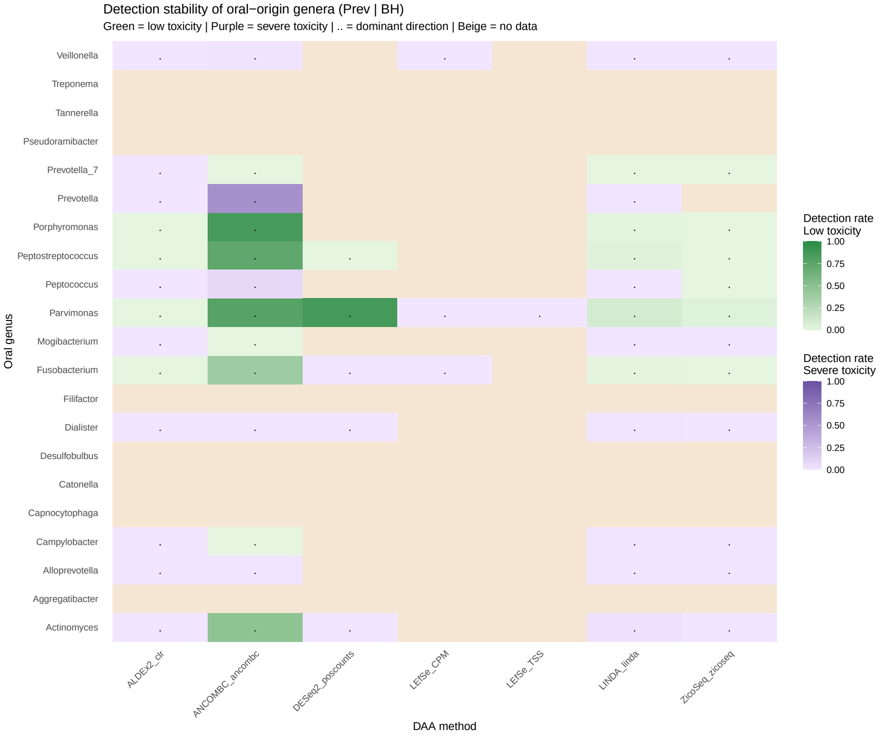

Fig. 5. Oral Bacteria Heatmap from primary dataset. Heatmap summarising the detection of selected oral bacterial taxa across different differential abundance analysis (DAA) methods using prevalence filtering and Benjamini–Hochberg (BH) multiple testing correction, a commonly recommended strategy for microbiome data analysis. Rows represent the selected oral taxa, and columns represent DAA methods. Cell colours indicate the detection rate (0–1), defined as the proportion of analyses in which a given taxon was identified as differentially abundant by each method. Additionally, taxa are annotated according to the toxicity group in which they were enriched, as classified by the respective DAA methods. (Referenced in manuscript as Supplementary Figure SF5).

Supplementary Figures (Confounder-adjusted models)

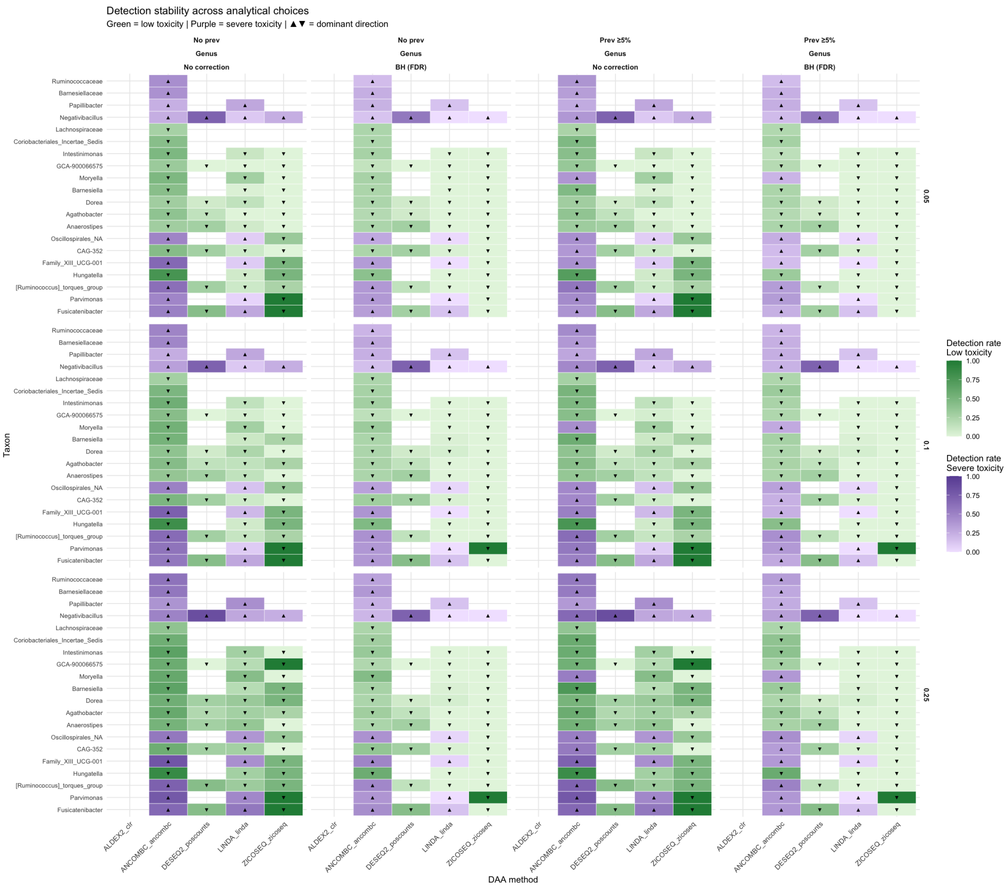

Fig. 6. Multi-approach heatmap in the primary dataset with covariates-adjustment. Heatmap summarising the detection of the top 20 bacterial taxa across multiple differential abundance analysis (DAA) methods under different analytical settings: two prevalence filtering strategies (no filtering vs. 5% filtering) and two multiple-testing correction approaches (none vs. Benjamini–Hochberg (BH) correction). Analyses were performed at both genus and ASV levels, a commonly recommended strategy in microbiome studies. Three significance thresholds (q-values) are shown on the right side of the plot to illustrate how detection varies across methods and thresholds. Rows represent the selected oral taxa, and columns represent the DAA methods. Cell colours indicate the detection rate (0–1), defined as the proportion of analyses in which a given taxon was identified as differentially abundant by each method. Taxa are additionally annotated by the toxicity group to which they were enriched, as classified by the respective DAA methods. White cells indicate taxa not detected under any condition by the respective method; faint-coloured cells reflect a detection rate of zero but an assignable direction of association.(Referenced in manuscript as Supplementary Figure SF6).

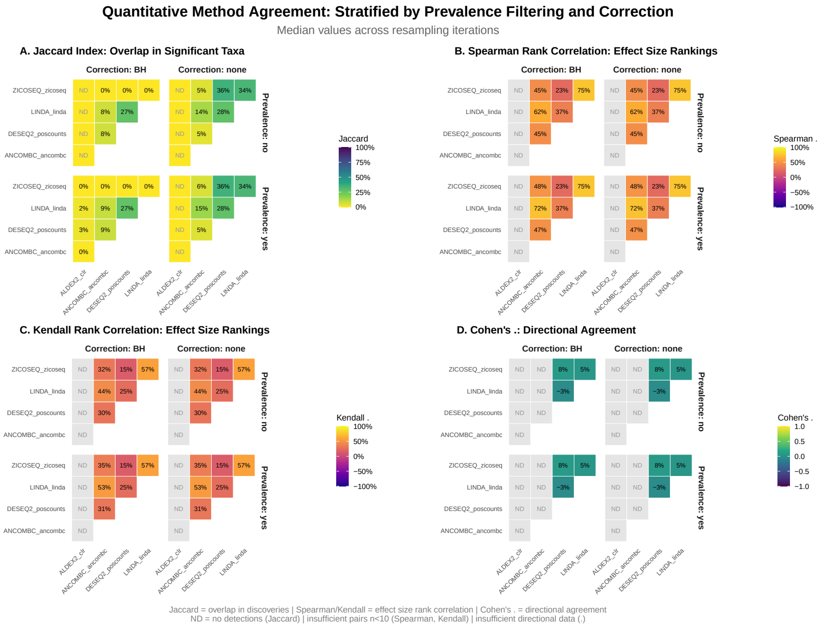

Fig. 7. Quantitative analysis of primary dataset with covariates. Heatmap summarising the detection of selected oral bacterial taxa across different differential abundance analysis (DAA) methods, with prevalence filtering and Benjamini–Hochberg (BH) multiple testing correction, in the validation dataset. Rows represent the selected oral taxa, and columns represent DAA methods. Cell colours indicate the detection rate (0–1), defined as the proportion of analyses in which a given taxon was identified as differentially abundant by each method. Additionally, taxa are annotated by the toxicity group to which they were enriched, as classified by the respective DAA methods. ND indicates that the metric could not be estimated for this stratum: no significant taxa were detected in either method (Jaccard); insufficient reliable feature pairs ($n \geq 10\%$) were available (Spearman, Kendall); or insufficient taxa with directional data were present (Cohen's κ). (Referenced in manuscript as Supplementary Figure SF7).

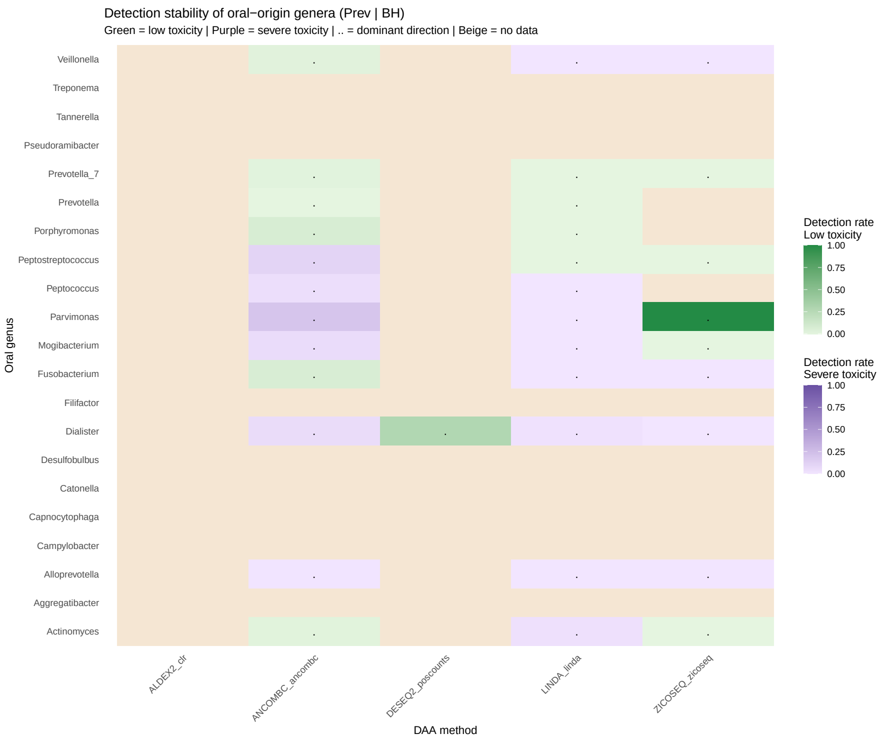

Fig. 8. Oral Bacteria Heatmap from primary dataset with covariates. Heatmap summarising the detection of selected oral bacterial taxa across different differential abundance analysis (DAA) methods, with prevalence filtering and Benjamini–Hochberg (BH) multiple testing correction, in the validation dataset. Rows represent the selected oral taxa, and columns represent DAA methods. Cell colours indicate the detection rate (0–1), defined as the proportion of analyses in which a given taxon was identified as differentially abundant by each method. Additionally, taxa are annotated by the toxicity group to which they were enriched, as classified by the respective DAA methods. (Referenced in manuscript as Supplementary Figure SF8).

Supplementary Figures (Validation Dataset)

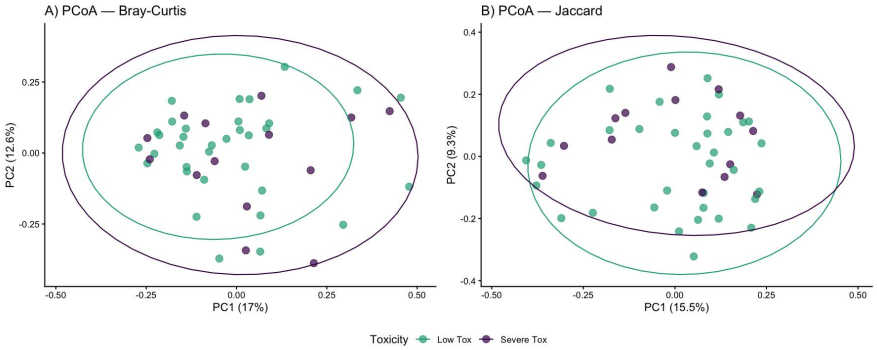

Fig. 9. The following figure illustrates the beta diversity plot of the external validation dataset. The Bray-Curtis distance was utilised to assess the dissimilarity between the two toxicity groups within this cohort. It is evident that there is a high level of diversity within the group. Furthermore, each toxicity group is associated with a colour: green for low and purple for severe. (Referenced in manuscript as Supplementary Figure SF9).

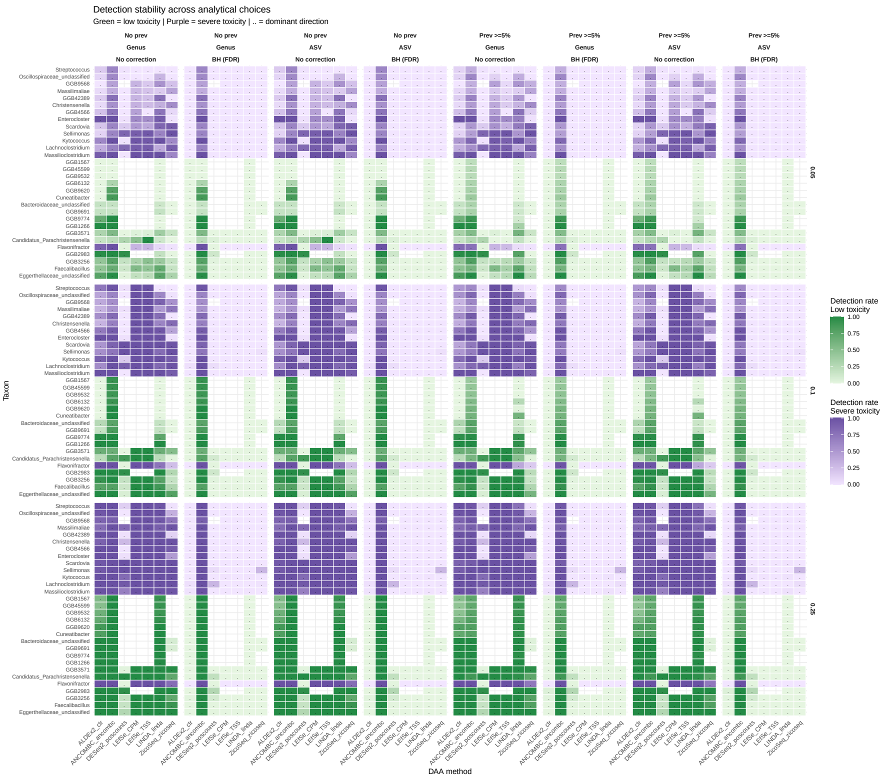

Fig. 10. Multi-approach heatmap from the external validation dataset. Heatmap summarising the detection of selected bacterial taxa across differential abundance analysis (DAA) methods, with prevalence filtering and Benjamini–Hochberg (BH) multiple testing correction, in the external validation dataset. Rows represent the selected oral taxa, and columns represent DAA methods. Cell colours indicate the detection rate (0–1), defined as the proportion of analyses in which a given taxon was identified as differentially abundant by each method. Additionally, taxa are annotated by the toxicity group to which they were enriched, as classified by the respective DAA methods. White cells indicate taxa not detected under any condition by the respective method; faint-coloured cells reflect a detection rate of zero but an assignable direction of association. (Referenced in manuscript as Supplementary Figure SF10).

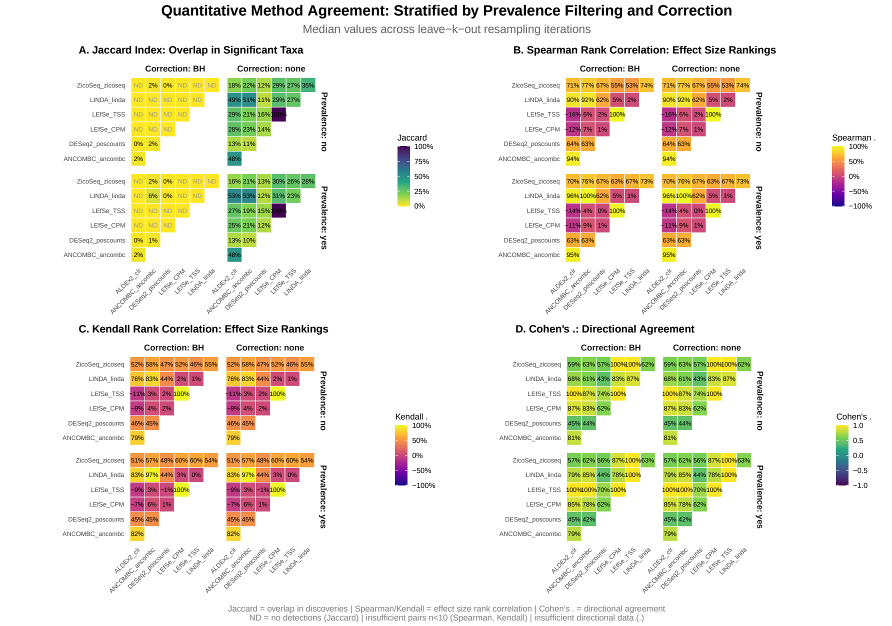

Fig. 11. Quantitative analysis of the external validation dataset. Rows represent the selected oral taxa, and columns represent DAA methods. Cell colours indicate the detection rate (0–1), defined as the proportion of analyses in which a given taxon was identified as differentially abundant by each method. Additionally, taxa are annotated according to the toxicity group in which they were enriched, as classified by the respective DAA methods. ND indicates that the metric could not be estimated for this stratum: no significant taxa were detected in either method (Jaccard); insufficient reliable feature pairs ($n \geq 10\%$) were available (Spearman, Kendall); or insufficient taxa with directional data were present (Cohen's κ). (Referenced in manuscript as Supplementary Figure SF11).

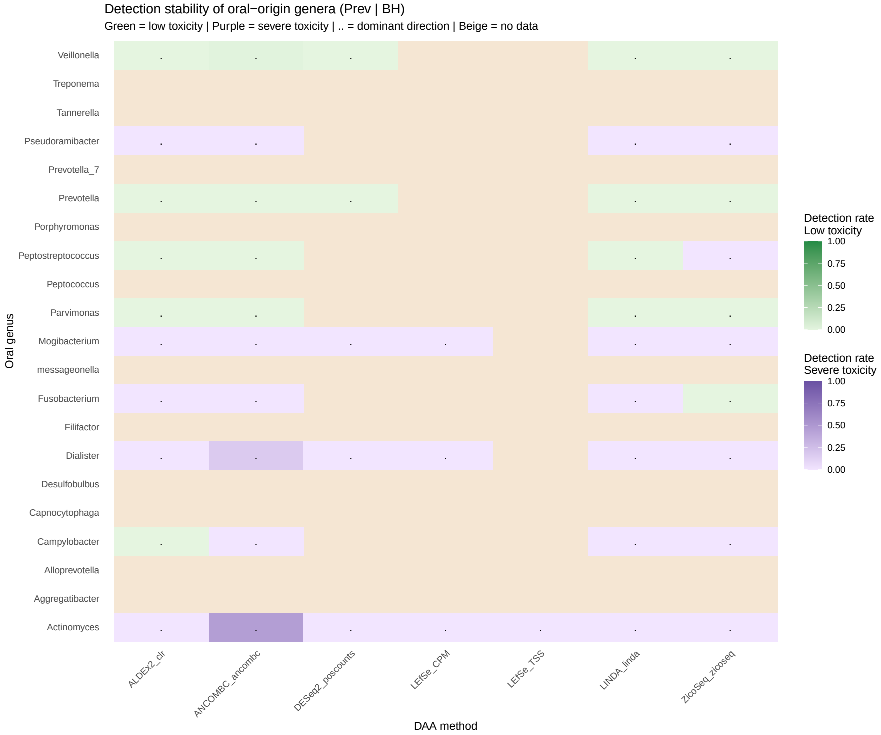

Fig. 12. Oral Bacteria Heatmap from external dataset. Heatmap summarising the detection of selected oral bacterial taxa across different differential abundance analysis (DAA) methods, with prevalence filtering and Benjamini–Hochberg (BH) multiple testing correction, in the external validation dataset. Rows represent the selected oral taxa, and columns represent DAA methods. Cell colours indicate the detection rate (0–1), defined as the proportion of analyses in which a given taxon was identified as differentially abundant by each method. Additionally, taxa are annotated by the toxicity group to which they were enriched, as classified by the respective DAA methods. (Referenced in manuscript as Supplementary Figure SF12).

References

1. Fernandes, A.D., Reid, J.N., Macklaim, J.M., McMurrough, T.A. (2014). Unifying the analysis of high-throughput sequencing datasets: characterizing RNA-seq, 16S rRNA gene sequencing and selective growth experiments by compositional data analysis. *Microbiome*, 2, 15. doi:10.1186/2049-2618-2-15
2. Lin, H., Peddada, S.D. (2020). Analysis of compositions of microbiomes with bias correction. *Nature Communications*, 11(1), 3514. doi:10.1038/s41467-020-17041-7
3. Love, M.I., Huber, W., Anders, S. (2014). Moderated estimation of fold change and dispersion for RNA-seq data with DESeq2. *Genome Biology*, 15, 550. doi:10.1186/s13059-014-0550-8
4. Segata, N., Izard, J., Waldron, L., Gevers, D., Miropolsky, L., Garrett, W.S., Huttenhower, C. (2011). Metagenomic biomarker discovery and explanation. *Genome Biology*, 12, R60. doi:10.1186/gb-2011-12-6-r60
5. Yang, L., Chen, J. (2022). A comprehensive evaluation of microbial differential abundance analysis methods: current status and potential solutions. *Microbiome*, 10, 130. doi:10.1186/s40168-022-01320-0
6. Zhou, H., He, K., Chen, J., Zhang, X. (2022). LinDA: linear models for differential abundance analysis of microbiome compositional data. *Genome Biology*, 23, 95. doi:10.1186/s13059-022-02655-5
